# CO_2_ Rise Directly Impairs Crop Nutritional Quality

**DOI:** 10.1111/gcb.70568

**Published:** 2025-11-14

**Authors:** Sterre F. ter Haar, Peter M. van Bodegom, Laura Scherer

**Affiliations:** ^1^ Institute of Environmental Sciences (CML), Leiden University Leiden the Netherlands

**Keywords:** crop quality, direct CO_2_ effect, food nutritional value, ionome changes, malnutrition, nutrient security, plant nutritional content, plant stoichiometry

## Abstract

Rising CO_2_ levels indirectly affect food availability (e.g., through climate shifts, biological consequences, and shifting socioeconomic systems). Less known is the direct effect of CO_2_ on nutrient availability by changing the plant stoichiometry. We help fill this gap, presenting a new methodology and creating the largest meta‐analysis on edible plant parts to date, including the most data (5324 entries covering 29,524 observation pairs), crops (43), and (anti)nutrients (32). The results show a pervasive elemental shift across a wide range of species as CO_2_ rises. Elements respond differentially, with zinc decreasing the most. Plants with not only a C3 but also a C4 photosynthetic pathway respond. Impacts vary by species and tissue type. This stoichiometric shift can lead to (worsening) malnutrition even in previously sufficient populations. Nutrient security is under threat even if food security remains adequate; food will become more caloric and less nutritious.

## Graphical abstract

This analysis studies the direct effect of CO_2_ rise on nutrient quality by synthesizing pre‐existing data on ~59,000 samples across 43 different food crops, where half of the plants were grown under elevated CO_2_ levels we are likely to experience in the coming decades. We observe nutrient decreases across a wide range of crops and nutrients, with the most pronounced effects for essential micronutrients such as zinc, iron, and protein. Our food is becoming increasingly caloric, less nutritious, and potentially more toxic, highlighting the importance of both food security and nutrient security.

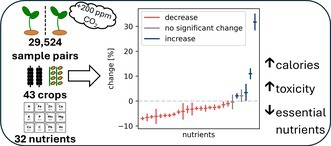

## 1 Introduction

We live in the Anthropocene, an epoch marked both by extreme climate change and global malnutrition (Giulia et al. 2020). Human activities raised atmospheric carbon dioxide (CO_2_) concentrations from ~280 ppm to ~425 ppm (Keeling et al. 2005), representing the most extreme increase in rate and amplitude of the past 3 million years (Gojon et al. 2023). CO_2_ rise–induced climate change is a cause and contributing factor to malnutrition (FAO et al. 2022). Climate change affects food security through a multitude of indirect effects by either reducing access (e.g., climate change–induced conflicts, price volatility) or by affecting production through climate shifts (e.g., extreme weather events, temperature increases) and/or biological consequences (e.g., pollinator declines, greater post‐harvest losses) (Myers et al. 2017). Less discussed is the direct effect of CO_2_ rise on malnutrition by changing nutrient availability through altering the plant's elemental nutrient composition. Initial postulations, dubbed the 'fertilization effect,' appeared hopeful; since the majority of plants use a C3 photosynthetic pathway and their growth is theoretically limited by CO_2_, climate change could increase primary plant biomass production, resulting in increased crop yields capable of feeding more people (Dahlman 1993). However, the effect of CO_2_ rise on nutrient quality is still understudied, leaving the focus on food security and limiting discussions on (micro)nutrient security.

Research on the direct impact of CO_2_ rise on crop nutrients is riddled with data gaps (Ebi et al. 2021; Ziska 2022) despite being on the research agenda since the 1970s (Strain 1978). The lack of studies has been called out before (Fangmeier et al. 2002; Loladze 2002; Lieffering et al. 2004; Giulia et al. 2020). Since 2002, the CO_2_ effect has been explicitly linked to hidden hunger (Loladze 2002). These warnings have amplified over time (e.g., Loladze 2014; Myers et al. 2014; Chumley and Hewlings 2020; Ben Mariem et al. 2021; Hu et al. 2022). The sparse research reveals a concerning trend, especially considering the problem's scale; while plants grow more rapidly under higher CO_2_ conditions, their protein and micronutrient content are lowered (e.g., Cotrufo et al. 1998; Loladze 2014; Myers et al. 2014; Taub et al. 2008; Medek et al. 2017), decreasing nutrient availability (Beach et al. 2019). A diet containing sufficient nutrients today could potentially be a nutritionally poor diet in the future due to the decreasing nutrient density in plant‐based foods. Few studies have quantified changing plant stoichiometries in future scenarios, and those that have were limited in the crops and nutrients analyzed (Högy et al. 2009; Loladze 2014; Myers et al. 2014). We use the term stoichiometry to describe our focus on elemental nutrient shifts, rather than the broader changes in chemical substances such as vitamins. To the best of the authors' knowledge, since then, no meta‐analysis considers more than the nitrogen response in edible parts of food crops (Medek et al. 2017) in all major plant‐based food groups. Smaller analyses have been conducted for legumes and vegetables (Scheelbeek et al. 2018), vegetables (Dong et al. 2018), rice (Hu et al. 2022; Kumar et al. 2023), wheat (Pleijel and Högy 2015), and cereals (Ben Mariem et al. 2021; Broberg et al. 2017). The two most well‐known analyses (Loladze 2014; Myers et al. 2014), while providing meaningful insights on patterns, compared the responses of the plant nutrients to ambient CO_2_ concentrations with limited regard for the experimental conditions. No analysis unifies the existing data, standardizes it (Toreti et al. 2020), and analyzes a wide range of elemental nutrients for edible parts of crops representing all major food categories with sufficient statistical power (Ebi and Loladze 2019). We fill this gap to better understand the impact of elevated CO_2_ levels on food and the overall implications for nutrient security by creating an updated database of CO_2_ effects on all major crops and their edible parts, incorporating available elemental response data and standardizing it to a reference baseline and an elevated CO_2_ level. This improves the accuracy of the modeled results, extends their generalizability, and sheds light on the complex factors underlying the variation in response rate. These studies are categorized and analyzed in a way that retains sufficient power to create confidence intervals for likely effects of an increase from 350 ppm, sometimes referred to as the last 'safe' level (Hansen et al. 2008), to 550 ppm, a point which will be reached by 2065 (Meinshausen et al. 2020) under SSP2‐4.5, a likely future given current and pledged policies (Hausfather and Peters 2020), and already before 2050 (Meinshausen et al. 2020) under SSP5‐8.5, a worst‐case scenario (Hausfather and Peters 2020).

## 2 Materials and Methods

### 2.1 Database Creation

The new database is an expanded dataset focused on edible parts that uses an older noteworthy dataset (Loladze 2014) as a base, adding in data(sets) from newly found literature. All previous entries (Loladze 2014) were filtered to match our inclusion criteria, reprocessed and checked, and extra (meta)data was added, resulting in some changes which consisted mostly of small value adjustments, fixing errant metadata, and/or including protein and phytate (Appendix S2). Another noteworthy paper (Myers et al. 2014) had its references consulted and added in a similar process. The reprocessing of their raw data, which was published later (Dietterich et al. 2015), can be seen as a recreation of their analysis, with similar but non‐identical results. Minor changes were made to their dataset to create consistent cultivar names with the rest of the database and include values listed as ranges. We elected to use the FACE wheat (*Triticum aestivium*) data directly from the original works (Fernando et al. 2012a, 2012b, 2012c; Fernando, Panozzo, Tausz, Norton, Fitzgerald, et al. 2014; Fernando, Panozzo, Tausz, Norton, Neumann, et al. 2014). A snowball search with the LENS.org search engine was conducted to update the combined database with newly published articles and to capture studies overlooked in previous meta‐analyses. This method was chosen because it finds a comparable amount of literature as a traditional search, but is more time‐efficient for a second‐generation literature review (Wohlin 2016). This was done in two phases: forward and reverse. The forward method is the first phase. All papers citing either well‐known paper (Loladze 2014; Myers et al. 2014) or their (Myers et al. 2014) raw data (Dietterich et al. 2015) are examined for inclusion. Any review or meta‐analysis was included in the second phase, a reverse snowball search. This is when the citations of the meta‐analyses and review papers were examined for further inclusion. This process was done iteratively until no new papers were identified.

The creation of the database from existing datasets (Loladze 2014; Dietterich et al. 2015; Myers et al. 2014) amounted to 1838 entries (containing replicates amounting to a total of 8823 observation pairs), along with a snowball literature search (Wohlin 2016) in a forward and reverse phase, yielding 69 articles with a total of 3486 data entries (covering 20,701 observation pairs). One thousand three hundred and thirteen articles were examined during the forward phase and identified ten meta‐analyses or review papers for the reverse snowball (Alae‐Carew et al. 2020; Ben Mariem et al. 2021; Doddrell et al. 2023; Dong et al. 2018; Hu et al. 2022; Kumar et al. 2023; Semba et al. 2022; Singer et al. 2020; Soares, Santos, et al. 2019; Toreti et al. 2020) (Table 1). Several papers had inaccessible or missing (supplementary) data (Jena et al. 2018; Köhler et al. 2019; Soares, Deuchande, et al. 2019; Soares et al. 2021; Broberg et al. 2017). These authors were individually contacted. Only one responded (Köhler et al. 2019). While the search method identified pre‐2014 papers not found or included by previous well‐known papers (Loladze 2014; Myers et al. 2014), they do not represent a large amount of data that could skew their conclusions. The snowball data represent a substantial portion of the dataset, covering new species and elements. The effect size was plotted against the number of replicates to test for publication bias (Figure S1) (Egger et al. 1997).

**TABLE 1:** The number of articles found and excluded during different steps in both the forward and reverse phases of the snowball search for inclusion in the database for further analysis.

	Dietterich	Loladze	Myers
*Forward snowball*			
Articles citing	63	301	949
Journal articles in English	49	245	702
Kept based on title	25	68	164
Kept based on abstract	17	51	111
Kept based on skim	9	41	65
Total found (forward)	115		
Excluded duplicates	−30		
Excluded: failed to meet criteria	−38		
**Articles included (forward)**	**47**		
*Reverse snowball*			
Review/meta‐analyses examined	10		
New articles with relevant titles examined further	64		
Excluded: unlocatable	−1		
Excluded: not in English	−8		
Excluded: did not discuss minerals	−32		
Excluded: combined factors	−2		
Excluded: multi‐generational	−1		
Excluded: review paper (no new data)	−1		
Articles found via non‐review article	3		
**Articles included (reverse)**	**22**		
**Total new articles included (forward and reverse)**	**69**		

The search was conducted in October and November of 2023. Only journal articles written in English were examined. We limited our search to commonly eaten parts of plants. This scoping discards previously identified data (Loladze 2014), but allows the analysis and interpretation to focus on the possible nutrient content effects relevant to consumption. Changes in non‐edible parts are still relevant for plant physiological studies, but are beyond the scope of this research. Inclusion criteria were edible parts of crops grown at two or more CO_2_ levels, direct measurements of one or more minerals at two or more CO_2_ levels, and reported results given as either absolute concentrations or relative change. Reasons for exclusion were multi‐generational tests, testing only non‐edible (portions of) crops, exposing only a part of the plant to CO_2_, uncontrolled and/or unsystematic CO_2_ application during the growing season, super‐elevated levels of CO_2_, and/or multiple factors combined per study where it was impossible to differentiate the direct effect of CO_2_ (e.g., testing ambient CO_2_ vs. elevated CO_2_ and ozone together). The focus was on mineral nutrients, so data on elements carbon and nitrogen were included only when presented along with data on minerals to examine the relationship between elements. Nitrogen is a linearly related proxy for protein (Myers et al. 2014), so (total/crude) protein was reported as protein along with nitrogen when given. Nitrogen proxy is calculated by using nitrogen when possible, and otherwise protein, but never both for the same data point. Antinutrient phytate was included when presented alongside mineral response data because of its effect on mineral absorption during consumption. Other biochemical substances were not included, and changes in their levels cannot always be represented by changes in minerals (Zhu et al. 2018). Only independent results were included, so if a paper recorded results at multiple time intervals for one experiment, only the latest results for the most mature edible plant parts were included. Multiple parts of the same plant were excluded, and only the (most commonly eaten) edible part was included. These data inclusion rules allowed for the greatest variety of data while still keeping the entries independent. Data were taken from tables, text, or Supporting Information where possible. WebPlotDigitizer was used to extract data from figures (Rohatgi 2022). Ambient CO_2_ levels were estimated using the Keeling Curve (Keeling et al. 2005) when they were not given by the study authors. Per entry, up to 31 pieces of (meta‐)data are recorded, including the calculated delta and natural logarithm of the response rate. Locations for FACE (free‐air CO_2_ enrichment) and open top chamber (OTC) experiments were taken from the text when possible, and otherwise, the research institute's location was used. Limited data samples precluded applying cofactors such as fertilizer application, but they are left within the database for future use. Cofactors include temperature, irrigation, sowing time, phosphorus application, nitrogen application, and ozone application.

### 2.2 Response Rate Linearization

Few authors have acknowledged or attempted to resolve the inconsistent experimental conditions. Linearizing the response rate by standardizing the dataset to a reference baseline and elevated CO_2_ level was used to solve the problem of population heterogeneity by accounting for the varying experimental conditions for the ambient and elevated CO_2_ levels. This method assumes that response heterogeneity is driven mainly by the variation in CO_2_ levels. While plants have a CO_2_ saturation point, previous research looking at the effect of CO_2_ rise on nutrient stoichiometry, especially on minerals, shows a linear response in the ranges considered here (Poorter et al. 2022; Pleijel and Högy 2015). The dataset records the delta (Equation 1) and the log response rate (Equation 2). The delta is defined as follows:

(1) $$\Delta =\frac{r_{E}-r_{A}}{r_{A}}\Leftrightarrow r_{E}=\Delta r_{A}+r_{A},$$

where

$$\begin{array}{l} r_{A}=\text{response}\;\mathrm{at}\;\text{ambient}\;{\mathrm{CO}}_{2}\\ r_{E}=\text{response}\;\mathrm{at}\;\text{elevated}\;{\mathrm{CO}}_{2} \end{array}$$

A is the ambient CO_2_ level and E is the elevated CO_2_ level. The natural logarithm is used as the response effect metric to reduce the bias from skewed data that are bound to a 100% decrease with a theoretically limitless increase (Hedges et al. 1999). It is defined as

(2) $$\ln\left(\frac{r_{E}}{r_{A}}\right)$$

To standardize the values to a given baseline ($r_{A^{*}}$) and elevated CO_2_ level ($r_{E^{*}}$), we use the formula for linear interpolation:

(3) $$y=y_{1}+\frac{\left(x-x_{1}\right)\left(y_{2}-y_{1}\right)}{\left(x_{2}-x_{1}\right)}$$

Substituting this for $r_{A^{*}}$ and $r_{E^{*}}$ in Equation (2) and using Equation (1) to rewrite it, we get:
(4)

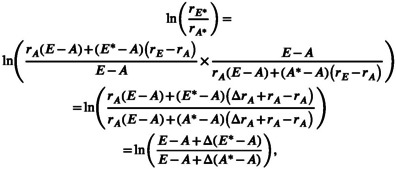

where

$$\begin{array}{l} A^{*}=\text{standardized ambient}\;{\mathrm{CO}}_{2}\;\text{level}\\ E^{*}=\text{standardized elevated}\;{\mathrm{CO}}_{2}\;\text{level} \end{array}$$

The adjusted log response results were converted back to percent change. All results are shown as percent change unless otherwise noted.

For the following analyses a baseline, $A^{*}$, CO_2_ level of 350 ppm was selected for two main reasons: one, 350 ppm is sometimes referred to as the last 'safe' level (Hansen et al. 2008) and secondly because it is within the range (310–455 ppm; average of 386.5 ppm) of the non‐adjusted ambient CO_2_ levels from the database. In shared socioeconomic pathway (SSP) scenario 1–1.9, 350 ppm is the stabilization and plateau point in 2150 after which only very slight further decreases take place. The elevated CO_2_ level was standardized to 550 ppm. Even though this threshold does not represent a physiological threshold for plant responses to CO_2_, it is an important societal threshold which is not reached by SSP1‐2.6 and SSP4‐3.4, but is exceeded in all other scenarios (in 2065 under SSP‐2.4.5, 2052 under SSP3‐7.0, 2060 under SSP4‐6.0, 2051 under SSP5‐3.4, and 2049 under SSP5‐8.5) (Meinshausen et al. 2020). The studies were conducted at varying levels of ambient and elevated CO_2_ (Figure S2). The effect of global CO_2_ rise is already reflected in the ambient CO_2_ levels; earlier experiments were conducted at levels closer to 350 ppm, while modern experiments were conducted at around 415 ppm. There is also a variation between studies on amounts of CO_2_ added, with the largest peak at 200 ppm, setting a precedent for setting 200 ppm as the added CO_2_ amount.

The majority of experiments were conducted above 550 ppm, so interpolation and not extrapolation were used to calculate the adjusted response. A linear response at reasonable levels of CO_2_ increase is an appropriate estimation (Poorter et al. 2022), but if the mineral saturation point is below or at the elevated experimental condition, our results are too conservative in estimating the effects of CO_2_ increase on the plant stoichiometry, as this study design linearly attributes the effect between the ambient and elevated levels, instead of between ambient levels and the elevated experimental condition. Either our estimate of the true effect is realistic or is too conservative and is therefore still appropriate for further analysis. This assumption was discussed (Ainsworth and Long 2005; Allen et al. 2020), modeled (Broberg et al. 2017; Pleijel and Högy 2015), and precedented by others (Scheelbeek et al. 2018; Medek et al. 2017).

### 2.3 Dataset Partitioning and Bootstrapping

To find possible factors that help explain the heterogeneity in responses, the dataset was split into meaningful categories based on evidence from the literature about expected influences. There are several possible factors with recorded (meta‐)data: between elements, photosynthetic pathway, experimental setups (i.e., FACE vs. OTC vs. greenhouse vs. chamber vs. tunnel; field vs. pot), plant parts (i.e., aboveground stems and shoots, reproductive parts such as fruits and seeds, and belowground parts such as roots and tubers), and taxonomy (e.g., family, genus, species). Different possible factors and combinations were included in the bootstrap analysis.

Rice grains were separated due to their unique growing conditions. Nitrogen proxy is used in the results because it has a larger sample size than protein or nitrogen alone. Differences between protein, nitrogen, and nitrogen proxy could be attributed to species‐specific effects and study type influence.

The sample and subgroups are never homoscedastic or normally distributed despite attempts at repairing this through outlier removal and log transformations, so a nonparametric method of analysis was used instead. Removing outliers is difficult to justify, and even the most aggressive methods were insufficient in creating a normal, homoscedastic sample. Using previously established methods (Loladze 2014), weighted bootstrapping with 10,000 replacements was used to calculate the 95% mean effect size confidence interval, the two‐sided *p*‐value with the null hypothesis being “no effect,” and the statistical power. The dataset was weighted by sample size, not variance, due to incomplete data (Wang, Loladze, et al. 2022). Experiments with more replicates had a higher chance of being (re‐)selected during bootstrapping with replacement. The mean and 95% confidence intervals were transformed back from the adjusted log response to the percent change. The *p*‐value was calculated by $p=\#\left[|Z^{*}|\;\geq \;|Z_{\mathrm{obs}}|\right]/10,000$, where # is the cardinality, *Z* is the test statistic, $Z^{*}$ is the *Z* calculated multiple times for the bootstrapped samples, and $Z_{\mathrm{obs}}$ was calculated from the dataset (Desgagné et al. 1998). Power is the chance the test will detect an effect size if present. The sample was shifted by the delta and then bootstrapped. The power is the fraction of these results that fall outside of the confidence interval for the original bootstrapped sample. The probability of a Type I error—a false positive—was set at α=0.05. When a sample is small, its variance can be much smaller than the population variance, resulting in an overestimation of power. Using the estimate of the population variance instead, when larger, is the more conservative approach. For small samples less than 20, the larger of the sample standard deviation or the standard deviation of the entire dataset was used for calculations. Results were categorized as low (< 0.4), medium (0.4–0.8), and high (> 0.8) power. This method is adapted from previous work (Loladze 2014). Both power and *p*‐values are useful to analyze together because an experiment with a small sample size can have a significant *p*‐value, but a low statistical power. Judging bootstrapped samples by both limits the analysis to powerful and significant results.

The response rate standardization measure applied here addresses the research gap in data measurement disparity (Toreti et al. 2020), increasing statistical power and significance. The larger dataset overcame the statistical power issues others struggled with, even when splitting the data. Power increased with data availability, so the calculated result is more likely to represent the true effect, illuminating the statistically significant shift of the plant stoichiometry and providing clarity on its magnitude and direction. The power was calculated for a 5% effect size, but many nutrients have an even larger effect size, suggesting an even higher true power. Compared to other work (Myers et al. 2014), our calculated effects are either similar or greater in magnitude, with narrower confidence intervals. The expanded dataset has enough data to observe previously undetectable differences. These results suggest that as the resolution and power increase, the measured effect remains either the same or increases in magnitude, and low‐powered analyses could still be used as conservative estimates of the true effect.

## 3 Results

### 3.1 Unprecedented Data Coverage

The database contains 5324 entries, representing observation pairs with potentially multiple replicates, from 109 articles (Table 2, S3) making it the largest known elemental database for edible parts of crops. Each article contributes less than 3% of the entries except for five studies: (16.7%) (Dietterich et al. 2015), (7.6%) (Ujiie et al. 2019), (4.6%) (Köhler et al. 2019), (3.7%) (Soares, Deuchande, et al. 2019), and (3.4%) (Jin et al. 2019). The largest dataset (Dietterich et al. 2015) is already clipped from the wheat studies which are listed separately but still comprises the results of five different studies on rice (7.0%), soybean (5.6%), peas (2.3%), maize (0.9%), and sorghum (0.9%). Not one experiment dominates the results, reducing experimental bias. Comparing the effect size across the number of replicates suggests either no or possibly only a small amount of publication bias (Figure S1). Our database covers 30 elements, plus protein and antinutrient phytate, which inhibits nutrient absorption. From here on, we collectively refer to them as nutrients. Iron, zinc, calcium, phosphorus, and potassium are the most well‐studied nutrients. Plants using the C3 photosynthetic pathway represent 96.5% of the observations, and the rest are C4 plants. Plants following a CAM photosynthetic pathway are unstudied in the context of plant stoichiometry responses to CO_2_ rise despite showing a response to increased CO_2_ (Drennan and Nobel 2000). These plants, which include pineapples, cacti, and agave, represent an extremely small portion of the global human diet.

**TABLE 2:** Summary of the data included in the database used for further analysis.

Data covered	Details
Entries	5324
Observation pairs	29,524
Photosynthetic pathway	C3 (96.5%), C4 (3.5%)
Plot types	field (64.0%), pot (36.0%)
Study types	free‐air CO_2_ enrichment (FACE) (58.0%), open top chamber (OTC) (17.4%; 11.9% in pot, 5.5% in field), indoor chamber (15.7%), greenhouse (8.3%), tunnel (0.5%)
Country (only outdoor experiments)	China (21.2%), USA (20.9%), Japan (19.4%), Australia (12.0%), Germany (10.7%), India (10.4%), Italy (3.5%), Brazil (0.7%), Sweden (0.4%), Portugal (0.3%), Philippines (0.1%), Denmark (0.1%), Finland (0.1%), UK (0.1%), Belgium (< 0.05%)
(Anti‐)nutrients covered	Fe (9.5%), Zn (9.4%), Ca (8.7%), P (8.5%), K (8.3%), Mn (8.3%), Mg (7.8%), Cu (7.2%), N (6.6%), S (4.6%), protein (3.7%), B (3.5%), Na (2.2%), phytate (2.0%), Mo (1.4%), C (1.3%), Ni (1.0%), Al (0.9%), Cr (0.8%), Cd (0.7%), Br (0.4%), Sc (0.4%), Si (0.4%), V (0.4%), As (0.3%), Cl (0.3%), Pb (0.3%), Se (0.3%), Sr (0.3%), Co (0.2%), Rb (0.2%), Ba (0.1%)
Tissue	reproductive (88.9%; 52.3% grain, 29.7% seed, 6.5% fruit, 0.2% pod, 0.1% fruit juice), aboveground (6.4%; 6.2% shoot; 0.2% stem), belowground (4.8%; 4.2% tuber, 0.6% root)
Crops	rice (28.5%), wheat (18.5%), soybean (18.1%), lettuce (3.5%), potato (3.4%), field pea (3.3%), bean (2.7%), tomato (2.6%), sweet pepper (2.4%), cucumber (1.6%), barley (1.5%), sorghum (1.4%), canola (1.0%), durum wheat (0.9%), maize (0.9%), peanut (0.9%), alfalfa (0.7%), faba bean (0.7%), foxtail millet (0.6%), millet (0.6%), radish (0.6%), chickpea (0.5%), mustard (0.5%), Chinese cabbage (0.4%), lentil (0.4%), oilseed rape (0.4%), brown bean (0.3%), carrot (0.3%), mustard spinach (0.3%), spinach (0.3%), turnip (0.3%), bok choy (0.2%), cabbage (0.2%), Chinese kale (0.2%), dill (0.2%), garden pea (0.2%), oat (0.2%), parsley (0.2%), snap bean (0.2%), sugar beet (0.2%), fenugreek (0.1%), broccoli (< 0.05%)
Families	Poaceae (53.2%), Fabaceae (28.1%), Solanaceae (8.3%), Brassicaceae (4.0%), Asteraceae (3.5%), Cucurbitaceae (1.6%), Amaranthaceae (0.7%), and Apiaceae (0.6%)

**FIGURE 1:**
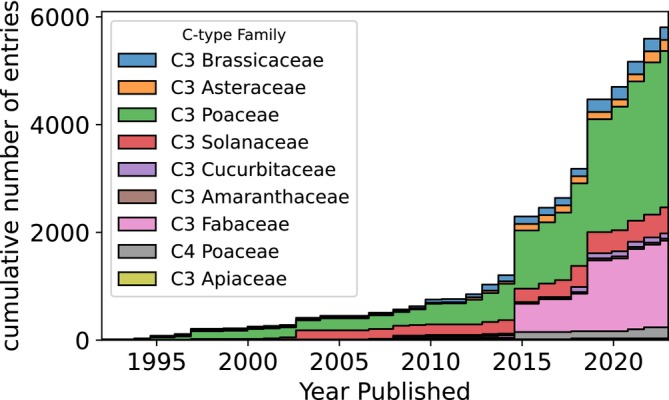
Growth of research on elevated CO_2_ over time. Shown as cumulative data entries published included in our database sorted by photosynthetic pathway and crop family.

Data publication took off in the 2010s (Figure 1) when free‐air CO_2_ enrichment (FACE) studies produced larger datasets including one notable dataset in 2015 (Dietterich et al. 2015). FACE experiments (58.0%) dominate the observations, followed by OTC studies (17.4%), taking place in 15 countries (Figure S3). The dataset contains the edible parts of 43 crops representing 225 cultivars from 28 genera contained in 8 families (Figure 1). Rice (28.5%) and wheat (18.5%), C3 grasses in the Poaceae family, are the most studied crops. Comparisons between cultivars are not yet possible except for rice, wheat, soybeans, and lettuce (Figures S5–S8). There is much less data on C4 plants, non‐grains, legumes other than soybeans, and non‐reproductive tissues. Maize, sugarcane and sugarbeet, cassava, oil palm, potatoes, and “oil crops (other),” are (largely) absent from studies despite being in the top 10 foods globally for calories provided (FAO 2024). The level to which the dataset can be split and remain powerful depends on the crop.

### 3.2 Differential Responses to CO_2_ Rise

**FIGURE 2:**
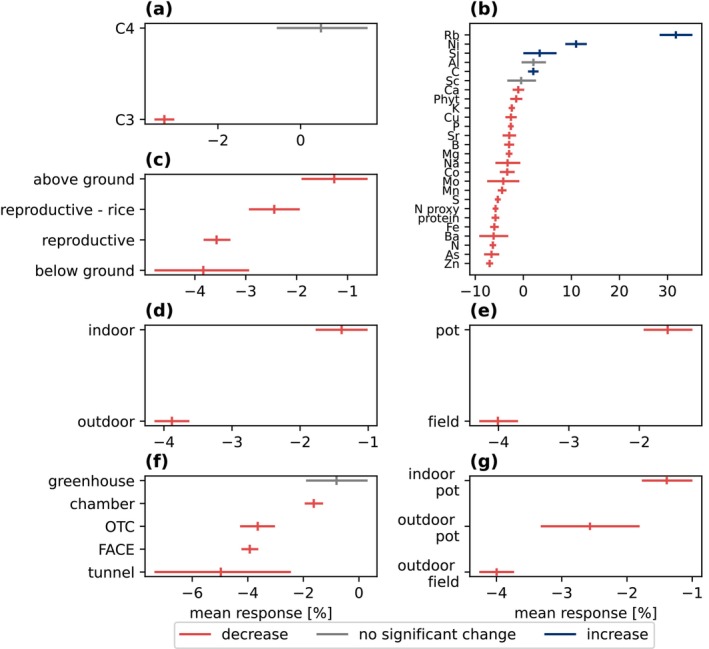
Percent change split per main factor for the entire dataset: (a) photosynthetic pathway (b) element and phytate (c) aggregated tissue type (d) experimental setup location (e) experimental setup growth container (f) experimental setup study type and container (g) experimental setup location and container. The arithmetic mean (tick) and the 95% confidence interval (line) are color‐coded by statistical significance interpretation and direction of change at α=0.05. Only high‐power results (> 0.8) are shown, which means that not all nutrients are shown in panel (b). N proxy means nitrogen proxy and includes both protein and nitrogen.

**FIGURE 3:**
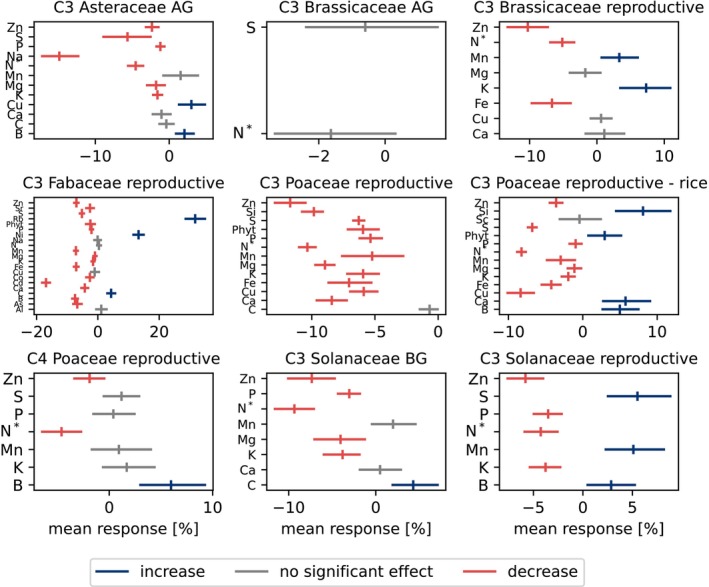
Percent change per element and phytate split per photosynthetic pathway, family, and tissue type. The arithmetic mean (tick) and the 95% confidence interval (line) are color‐coded by statistical significance interpretation and direction of change at α=0.05. Only high‐power results (> 0.8) are shown. BG stands for ‘belowground’ and includes roots and tubers. Reproductive contains all fruits, seeds, grains, and pods. $N^{*}$ means nitrogen proxy and includes both protein and nitrogen.

**FIGURE 4:**
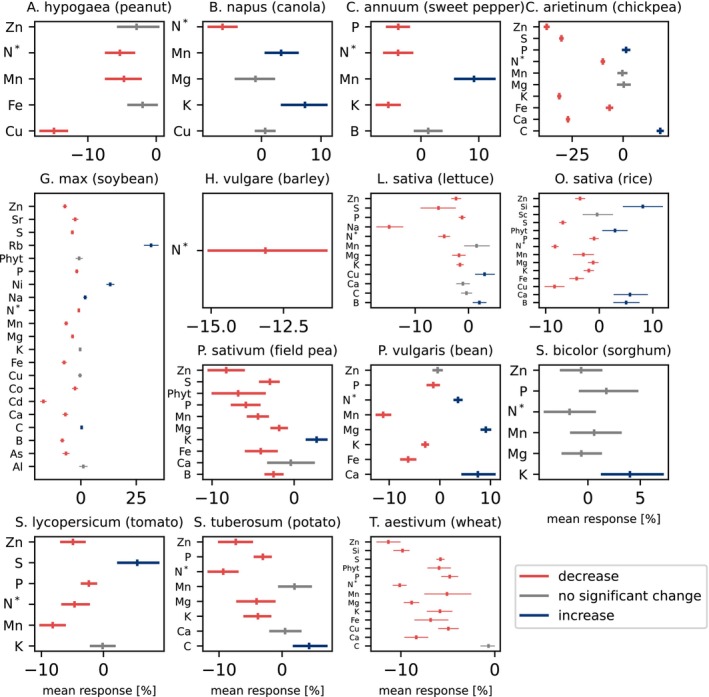
Percent change per element and phytate for species. The arithmetic mean (tick) and the 95% confidence interval (line) are color‐coded by statistical significance interpretation and direction of change at α=0.05. Only high‐power results (> 0.8) are shown. $N^{*}$ means nitrogen proxy and includes both protein and nitrogen. All species are C3 plants except C4 plant *S. bicolor* .

The dataset has a significant (*p*‐value < 0.001) mean decrease of 3.2% (−3.4%, −3.0%). As statistical power increases, the systemic shift in the plant stoichiometry emerges. Wide confidence intervals, usually in the low‐power region (power < 0.4), are due to noise and data limitations (Figure S4). The following analysis considers only high‐power statements unless otherwise stated. Several factors affect the CO_2_ response, including photosynthetic pathway, nutrient, tissue type, and study design (Figure 2), as well as taxonomic classification (Figures 3 and 4).

Photosynthetic pathway responses differ; C3 plants have a mean decrease, and C4 plants show no significant change at the aggregated level (Figure 2). Examining per nutrient and within families shows that C4 plants are also affected. Despite a weaker response than C3 plants, C4 Poaceae grasses also shift (negative: nitrogen, zinc; positive: boron) (Figure 3). C4 plant *S. bicolor* (sorghum) experiences a 4% increase in potassium (Figure 4).

There is considerable response rate variation between nutrients. 77% of nutrients with sufficient power (> 0.8) decrease and 15% increase (α<0.05) (Figure 2), ranging from a mean increase of 32% for rubidium to a 7.1% decrease in zinc in C3 plants and a mean increase of 6.0% for boron and 4.6% decrease in protein in C4 plants. Chickpeas ( *Cicer arietinum* ) have the largest powerful and significant mean change of a single nutrient (zinc: −37.5%) (Figure 4). Within the same family and species (Figures 3 and 4), there is considerable variation between nutrients.

Responses also differ per tissue type. The main differences between tissues appear to be driven by the function. We grouped these parts as “belowground” (roots and tubers), “reproductive” (grain/seed, fruit, pod), and “aboveground” (shoots and stems). Belowground parts decreased the most, followed by reproductive tissues, then aboveground parts.

Study type also has an effect. Outdoor OTC and FACE experiments (which are not significantly different from each other) show a three times larger mean decrease than indoor chamber and greenhouse experiments (which are not significantly different from each other), and per nutrient, outdoor experiments often show a similar or increased magnitude change.

We see a similar response difference for plants grown indoors and outdoors as we observe between plants grown in pots versus in fields, where plants grown in a field respond significantly more negatively than those in a pot. Distinguishing between both of them simultaneously still shows a significant response difference, where overall plants grown outdoors in a field respond more severely than outdoors in a pot or indoors in a pot, and all three groups are significantly different from each other. We see significant differences between OTC pot and field experiments for potatoes ( *Solanum tuberosum* , Figure S9), but we have too little power to analyze this per cultivar, which can also play a role. For lettuce ( *Lactuca sativa* , Figure S8) in chamber experiments, cultivars 'Rex' and 'Rouxai' show significant differences in magnitude for some elements (B, K, Mg, Zn), and sometimes even the opposite effect (Ca, Mn, P, S). Overall, cultivars tend to be in agreement over the direction but not always the magnitude. It is not yet possible to compare cultivars across study types. The response rates for different cultivars per study and plot type are shown in the SI for rice, wheat, soybeans, and lettuce (Figures S5–S8).

## 4 Discussion

### 4.1 Plants Respond Unevenly

Although the complex chemical–environment interactions are still under study (Ziska 2022) and we cannot yet precisely predict the response, we can accurately state that the plant stoichiometry is changing under rising CO_2_ levels, generally negatively affecting their nutrient levels. We can even quantify responses for specific crops and nutrients, extensively validating skeptically received previous predictions (Loladze 2002) and observations (Loladze 2014; Myers et al. 2014; Pleijel and Högy 2015; Broberg et al. 2017; Scheelbeek et al. 2018; Al‐Hadeethi et al. 2019; Chumley and Hewlings 2020; Ainsworth and Long 2021; Ben Mariem et al. 2021; Hu et al. 2021, 2022; Jayawardena et al. 2021; Kumar et al. 2023) that the plant stoichiometry is changing. Our results support what others have previously predicted (Loladze 2002) and found (Soares, Santos, et al. 2019; Ziska 2022; Kumar et al. 2023); factors beyond carbon dilution are involved in the response. We see a differential nutrient response (Figure 4), possibly due to their different functions (Broberg et al. 2017; Ebi et al. 2021) across all analysis levels (Figures 3 and 4). Smaller studies found similar results (Pleijel and Högy 2015; Ziska 2022; Myers et al. 2014), although we are among the first to show the predicted (Loladze 2002) increase in some nutrients. We need to further study the elemental saturation point, the response of unstudied nutrients, and plant stoichiometry at even higher CO_2_ levels. These will be important avenues of research to understand how food nutritional values will change over time. More data are needed on different nutrients and plants worldwide, but the cost of data collection is a barrier (Toreti et al. 2020).

### 4.2 C3 and C4 Plants Both Respond

The direct effect of CO_2_ rise from 350 ppm to 550 ppm downshifts the plant stoichiometry with negative consequences for the nutritional value. C3 and C4 plants have separate photosynthetic pathways, so logically they would respond differently to elevated CO_2_ levels. Research suggests that C4 plants are less responsive to CO_2_ rise than C3 plants, which represent 90% of plant species globally (Cotrufo et al. 1998; Ziska 2022) and 96.5% of published data (Figure 1; Table 2). The fertilization effect on C3 plants will result in a higher yield (Gojon et al. 2023), suggesting that C3 crops should be preferentially grown. However, if C4 plant stoichiometry is less affected by CO_2_ rise, C4 crops should potentially be selected for instead, and C4 photosynthetic mechanisms should be bred into C3 crops (Jobe et al. 2020). Others found no significant effect on C4 plant stoichiometry, attributing this to limited data unable to detect effect sizes less than 5% (Loladze 2014). While we observe an insignificant mean effect for C4 plant stoichiometry, splitting the data per nutrient shows a clear negative shift in nitrogen and zinc and a positive shift for boron. C4 plants provide critical nutrients in foods such as maize, millet, and sorghum. Maize and sugar (C4 sugarcane and C3 sugarbeet combined) are the third and fourth largest contributors (FAO 2024) to global calorie supply, but make up less than 1% of the data. It would be an oversimplification to assume that CO_2_ rise–induced changes in their nutrient content will be negligible. This idea needs to be reconsidered and tested further. If CO_2_ fertilization is used to inform decision‐making on how to select for or modify crops, their nutrient response should be taken into account and the effect of CO_2_ rise on both C3 and C4 crops should be better known. Such decision‐making is complicated by the highly species‐dependent nature of the effects, meaning that the effect CO_2_ rise will have on diets is not only dependent on the percentage of C3 foods consumed but also on the species composition.

### 4.3 Malnutrition Will Increase

The majority of nutrients responded negatively, suggesting that crops overall are becoming less nutritious, which threatens nutrient security. This mean effect is −3.2% for all nutrients combined but reaches up to a mean 37.5% reduction for zinc in chickpeas and a 31.7% increase in rubidium in soybeans. Multi‐generational experiments are limited, so long‐term effects are not yet well understood, but so far they show that the nutrient quality decrease is even larger than in single‐generation experiments (Li et al. 2019). Rice is the primary staple crop for over half of the world's population, and another 2.5 billion people are dependent on wheat (Ebi et al. 2021). Both show significant decreases in essential nutrients such as protein (proxied by nitrogen), zinc, and iron. Decreasing nutritional value can have devastating health consequences, contributing to (further) malnutrition, including in previously sufficient populations, by reducing nutrient availability even if food availability remains constant. It can contribute to hidden hunger, where people have sufficient food calorically but insufficient nutrients. The effect this will have in the context of a total diet on different sub‐populations or their physical health is a nascent avenue of research but should not be underestimated.

Research focuses on essential nutrients (e.g., zinc, iron) needed by both plants and humans in limited species. More research is needed on essential nutrients in other species. Additionally, although all elements essential for plants are essential for humans, the reverse is not true (Ebi et al. 2021; Loladze 2024). Nutrients non‐essential for plants can be essential for humans (Huang et al. 2020), and their response is not being sufficiently researched. Of the 10 elemental nutrients essential for humans but not for plants, seven elements (arsenic, cobalt, chromium, selenium, silicon, sodium, vanadium) are sparsely researched. Two of these, sodium and chromium, have more than two dozen data entries, but this still pales in comparison to the more than 400 entries each for other minerals such as iron, zinc, calcium, phosphorus, potassium, manganese, and magnesium. The other three minerals (iodine, fluoride, tin) are completely unstudied. Phytate, an anti‐nutrient that blocks the absorption of essential minerals, is limited in data to a few species despite being relevant in understanding the nutritional uptake of different diet compositions (Myers et al. 2014). There is a discrepancy between research needs and measurements (Toreti et al. 2020; Ebi et al. 2021), with non‐essential and toxic elements in all species being understudied. The dataset is insufficient to analyze toxic nutrients that can have negative health impacts. Mineral elements benign to plants can be harmful to humans, and the changing stoichiometry could shift these balances unfavorably. Heavy metals might increase; our results showed increases for chromium, lead, and nickel. Lead showed an average 29.3% (15.1%; 45.8%) increase (*p*‐value < 0.005, power = 0.12), increasing up to 170% (*p*‐value < 0.005, power = 0.0639) in wheat. A significant result despite low power implies a very strong effect, hinting at possible unintended shifts in the plant stoichiometry that are missed by current research focused on essential nutrients. Considering the severe health effects increasing consumption of these minerals can have (Balali‐Mood et al. 2021), non‐essential minerals warrant receiving more attention or else we will bear the unintended consequences of a shifting plant stoichiometry with potentially devastating health consequences. Additionally, we limited our analysis to chemical elements rather than biochemical substances such as vitamins (Zhu et al. 2018) and carotenoids (Loladze et al. 2019) which also show a CO_2_ response. While some suggest there may be a correlation between CO_2_‐induced changes in carbon and nitrogen levels and the levels of different vitamins, changes in secondary compound levels cannot yet be represented by changes in levels of primary elements (Zhu et al. 2018). Further research on specific biochemical substances is therefore warranted.

### 4.4 Interexperimental Comparisons Face Limitations

Looking at our results in Figure 2, it is tempting to say that outdoor experiments respond more strongly than indoor experiments, and similarly that field experiments respond more strongly than pot experiments. Others have previously discussed how to compare results from different experimental methods (e.g., McLeod and Long 1999; Lieffering et al. 2004; Ainsworth and Long 2005, 2021; de Graaff et al. 2006; Taub et al. 2008; Ainsworth 2008; Bunce 2011, 2016; Broberg et al. 2017; Allen et al. 2020). Previously found differences between experiments, for example between FACE/OTC experiments and chamber experiments, were likely due to the amount of CO_2_ added (Ainsworth and Long 2005) or by comparing effects of added CO_2_ versus ambient and elevated CO_2_ (Bunce 2016), and a cultivar effect has also been noted (Ainsworth 2008; Bunce 2016; Allen et al. 2020). The first experiments were conducted indoors (greenhouses, chambers), and in response to concerns that the growing conditions were not realistic, later outdoors (tunnels, OTCs, FACE experiments). While FACE experiments may grow under more realistic conditions, they face their own set of limitations which can lead to underestimating the effect (Bunce 2016). It is still unknown which experimental setup produces the most realistic results. Nevertheless, we have statistically powerful and significant results that show a generally concerning trend for crop nutrient content, and this result holds even when sub‐dividing the groups. This result is concerning, especially considering the debate suggesting that all experimental methods, for varying reasons, are conservative estimates, implying an even larger shift. Overall, we find that FACE and OTC results are not significantly different from each other once adjusting for CO_2_ levels. If OTC experiments can provide results of similar quality at a lower cost, this could encourage the required further data collection. Similarly, if we continue to observe that indoor experiments tend to be more conservative than outdoor experiments, it would still be useful to expand these types of experiments as a lower bound of possible change. Indoor experiments tend to be cheaper and are more widely accessible. We need more experiments under broader conditions to better understand the role of the species‐ and nutrient‐specific response if we want a chance at mitigating these effects. Discarding experimental results purely based on assumptions that a certain method is inferior for being too conservative or unrealistic would do a disservice to this cause.

### 4.5 Future Research Needs to Include Cofactors

Time of sowing (Fernando, Panozzo, Tausz, Norton, Fitzgerald, et al. 2014), temperature (Zhu et al. 2023), resource supply (Piñero et al. 2017), and ozone application (Allen et al. 2020) are known to alter plant responses. Cofactors were included in the database in hopes that they can be of future use. Most of what we know about how plant responses vary based on experimental type considers yield. We know even less about the response curves for nutrient uptake (Poorter et al. 2022). Future research on diverse plants and cultivars at varying CO_2_ levels and under different experimental conditions are needed. These data gaps must be filled before we can start selecting to maintain nutritional integrity in a higher CO_2_ world, let alone understand the impacts of dietary composition on nutrient vulnerability and how to reduce the impacts in a culturally and economically appropriate way. Future study can build upon this and previous study (Loladze 2014) to include non‐edible plants for a more universal understanding.

### 4.6 Emission Reduction Preserves Nutritional Quality

The CO_2_ rise–induced plant stoichiometric downshift is a concern for nutrient intake, but we can avoid major nutrient losses by lowering our emissions. At present, we are at 425.2 ppm (Keeling et al. 2005), putting us at 37.6% of the way through the modeled change in atmospheric CO_2_ concentration, where we are already experiencing lowered levels of plant nutrition due to CO_2_ rise. By 2100, global CO_2_ projections are between 393.5 and 1135.2 ppm, depending on the pathway. Under pathway SSP1‐1.9 in line with the 1.5°C climate target, emissions would peak in 2041 at 440.2 ppm, meaning a reduced nutrient quality of 45.1% compared to the 350 ppm baseline. As emissions lower, we would reach 393.5 ppm by 2100 (Meinshausen et al. 2020) where we would still experience reduced food nutrient composition due to CO_2_ levels, but it would allow us to avoid 78% of the effect up to the 550 ppm modeled here. By 2149, the emissions would drop below the ambient baseline used in the model, bringing food back to its original nutritional values. SSP1‐2.6 would peak in 2063 at 474.0 ppm (Meinshausen et al. 2020) with 62% of the modeled effects and SSP4‐3.4 would peak in 2079 at 490.6 ppm (Meinshausen et al. 2020) with 70% of the modeled effects. The other SSPs will exceed the modeled changes in 2065 (SSP‐2.4.5), 2052 (SSP3‐7.0), 2060 (SSP4‐6.0), 2051 (SSP5‐3.4), and 2049 (SSP5‐8.5) (Meinshausen et al. 2020). This highlights the benefits of action: the lower the CO_2_ emissions, the lower the nutrient loss due to the CO_2_ effect.

### 4.7 Implications Also Concern Industrially Grown Food

The lower nutrient stoichiometric response to increased CO_2_ rise is also a concern for industrially grown food in CO_2_‐enriched greenhouses. To compete in the global market and to keep up with increasing urbanization, we expect intensive agriculture will become more important for food supply (Nemali 2022), which increasingly uses enriched CO_2_ to raise productivity (Wang, Lv, et al. 2022). Greenhouses can aid in improving dietary quality by increasing food diversity and availability, but the nutrient concentration of these foods should be included as an additional perspective. Our results show that food grown under higher CO_2_ regimes may suffer from mineral loss. Agronomic management could benefit from including these perspectives to mitigate (some of) these effects. Our food system would benefit from centering nutrient security alongside increasing food security to make high‐quality diets a reality for a broader portion of the population.

## Author Contributions

**Sterre F. ter Haar:** conceptualization, data curation, formal analysis, methodology, visualization, writing – original draft, writing – review and editing. **Peter M. van Bodegom:** conceptualization, methodology, supervision, writing – review and editing. **Laura Scherer:** conceptualization, funding acquisition, methodology, supervision, writing – review and editing.

## Conflicts of Interest

The authors declare no conflicts of interest.

## Supporting information


**Appendix S1:** Supporting figures.

**Appendix S2:** Explanation of changes made between prior databases (Loladze 2014; Myers et al. 2014; Dietterich et al. 2015) and ours for transparency over changes we made and to prevent future users from double‐counting entries.

**Data S1:** The full citation of all the data sources used in the input database.

## Data Availability

The data and code that support the findings of this study are openly available in Zenodo at http://doi.org/10.5281/zenodo.17274164.
