# Supplementary material for: CO2 Rise Directly Impairs Crop Nutritional Quality
Source: Glob Chang Biol. 2025 Nov 14;31(11):e70568. doi: 10.1111/gcb.70568 (PMC12616468; doi:10.1111/gcb.70568)
Supplement: Supplementary file 1 — Appendix S1: Supporting figures. [file GCB-31-e70568-s003.pdf]

CO_2 Rise Directly Impairs Crop Nutritional Quality
Appendix S1: Supporting Figures

S.F. ter Haar[1,*], P.M. van Bodegom[1], and L. Scherer[1]

[1]Institute of Environmental Sciences (CML), Leiden University, Leiden, The Netherlands
[*]email: s.f.ter.haar@cml.leidenuniv.nl

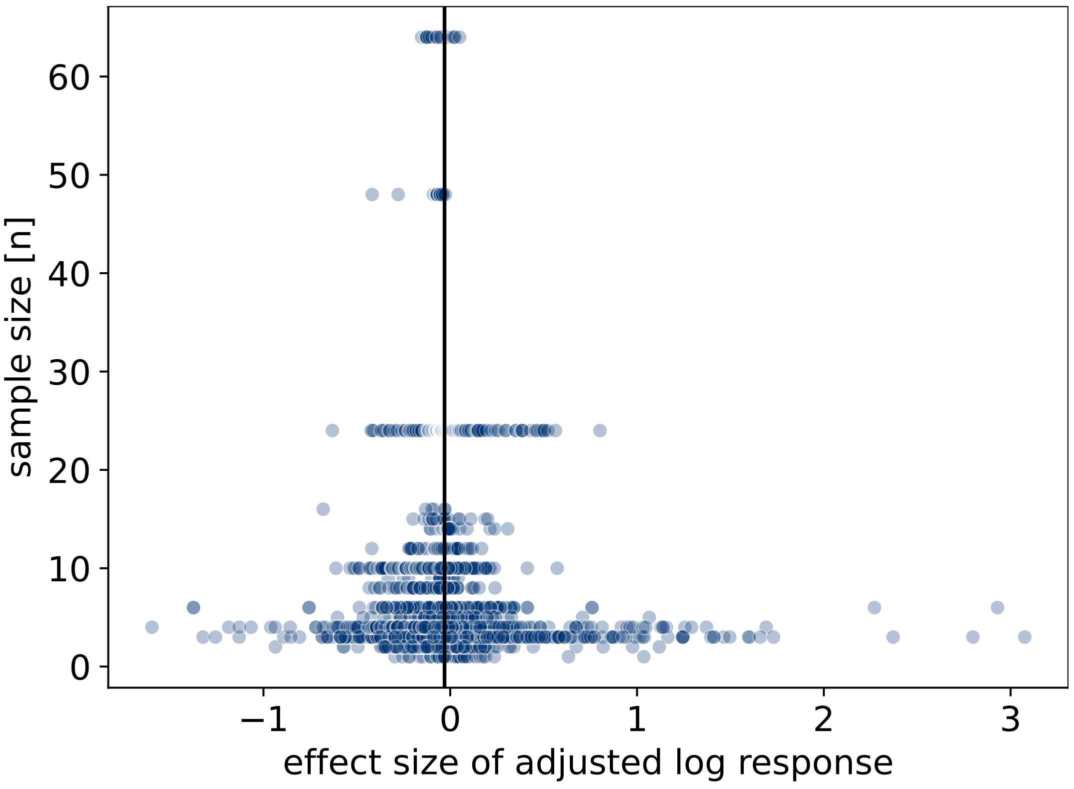

Figure S1. Publication bias of the whole adjusted dataset. A funnel plot tests for publication bias in the whole dataset. The black line is the mean effect of the population. The term 'effect size of adjusted log response' is equivalent to '$\ln(E^*/A^*)$' and refers to the natural logarithm of the response at the standardized elevated CO_2 levels divided by the response at standardized baseline ambient CO_2 levels. The resulting scatter plot is funnel-shaped, wider at the bottom, and narrows as the sample size increases. The points are mostly symmetrical about the mean, with some outliers on the right-hand side.

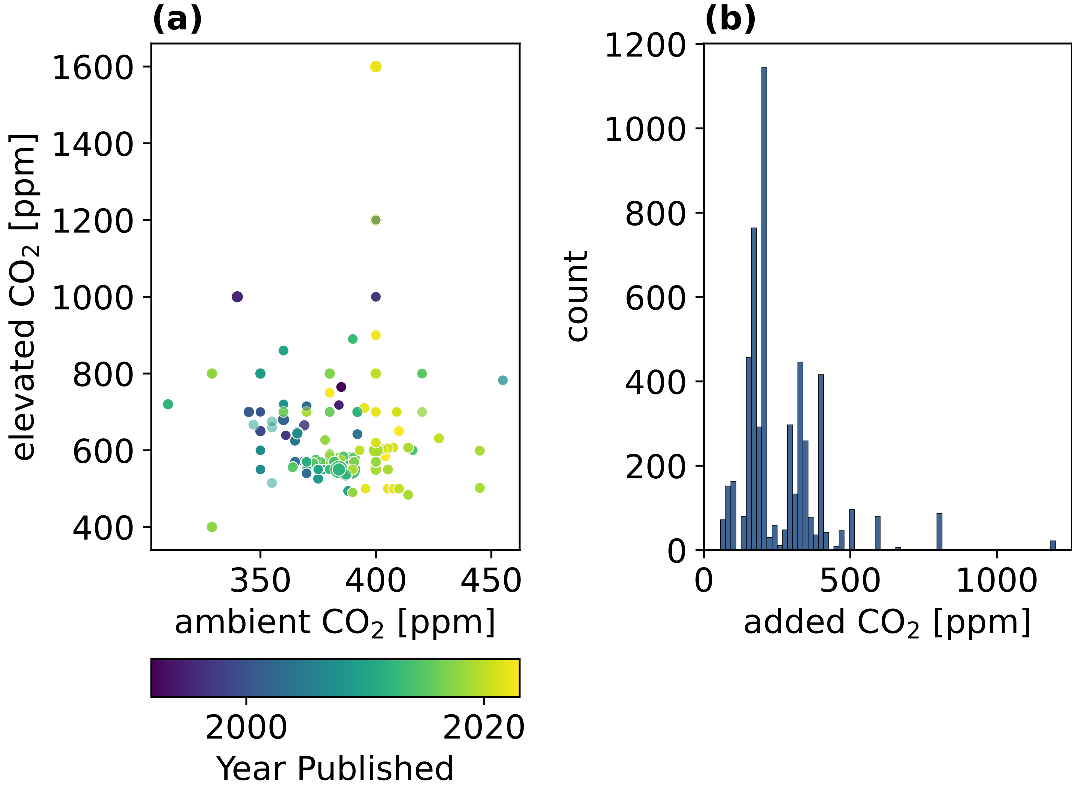

Figure S2. **Experimental distribution of CO_2 levels in the database.** a) Scatterplot of ambient versus elevated CO_2 levels and b) histogram of added CO_2 levels. For panel (a) the points are sized based on their total number of replicates.

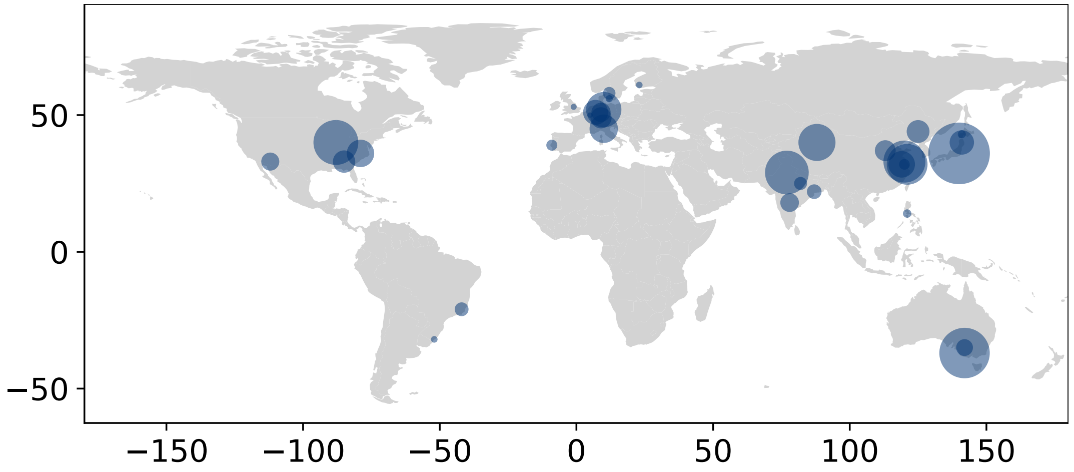

Figure S3. **Study locations of outdoor FACE and OTC experiments.** Nearby locations are clustered together. The size of the dots are linked to the amount of data entries.

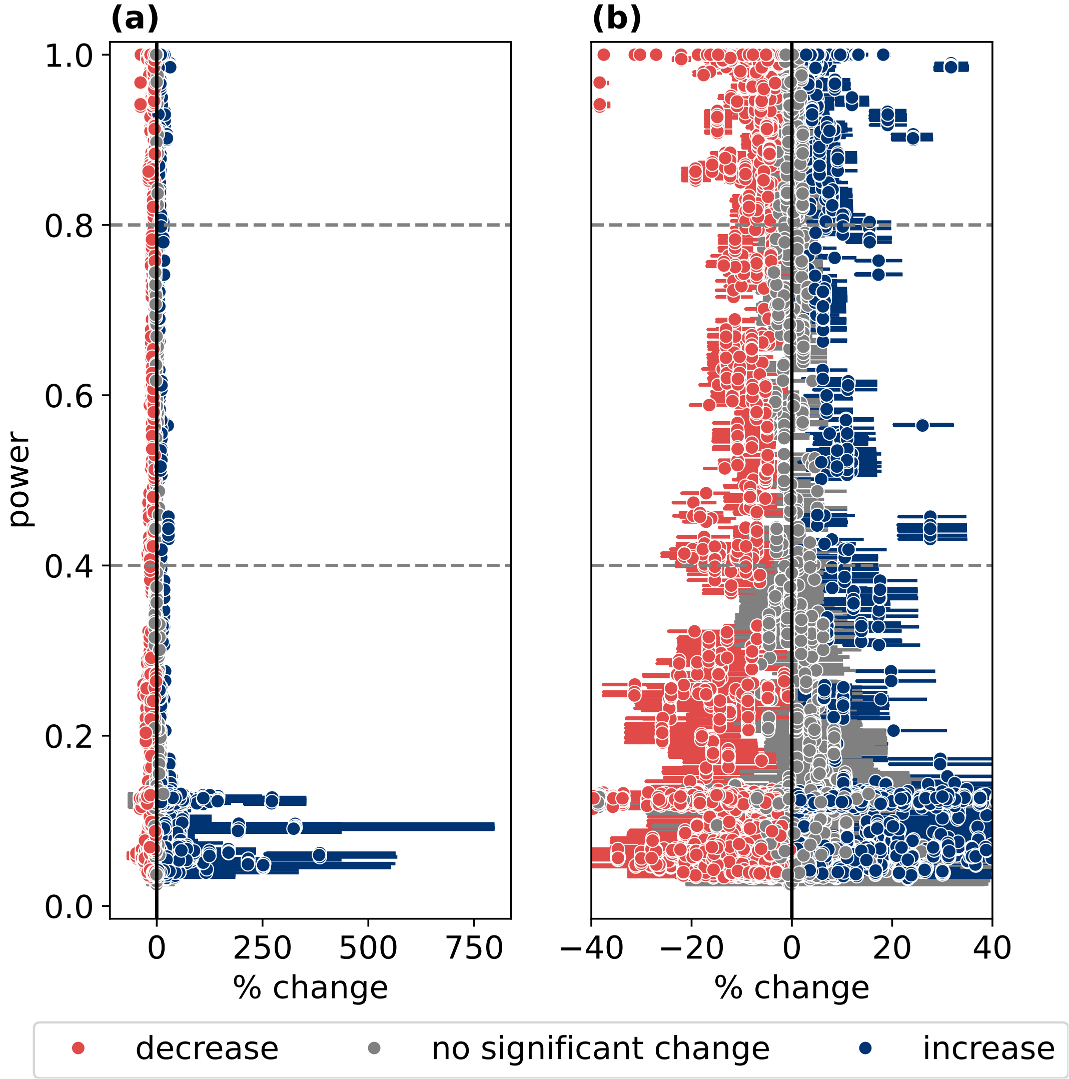

Figure S4. Percent change adjusted log response to a 200 ppm CO_2 increase. Panel (b) is a zoomed-in version of panel a to show the effect near x=0. All results are plotted for the weighted bootstrapping results for the entire database, per photosynthetic pathway, element, tissue type, aggregated tissue type, and study design, and for varying combinations of the above factors, including splitting by indoor/outdoor experiments. Points denote the arithmetic means and are connected to lines denoting the 95% confidence interval. The significance level is set to $\alpha = 0.05$. Horizontal dashed lines denote the divisions between the low (0-0.4), medium (0.4-0.8), and high (0.8-1) power regimes.

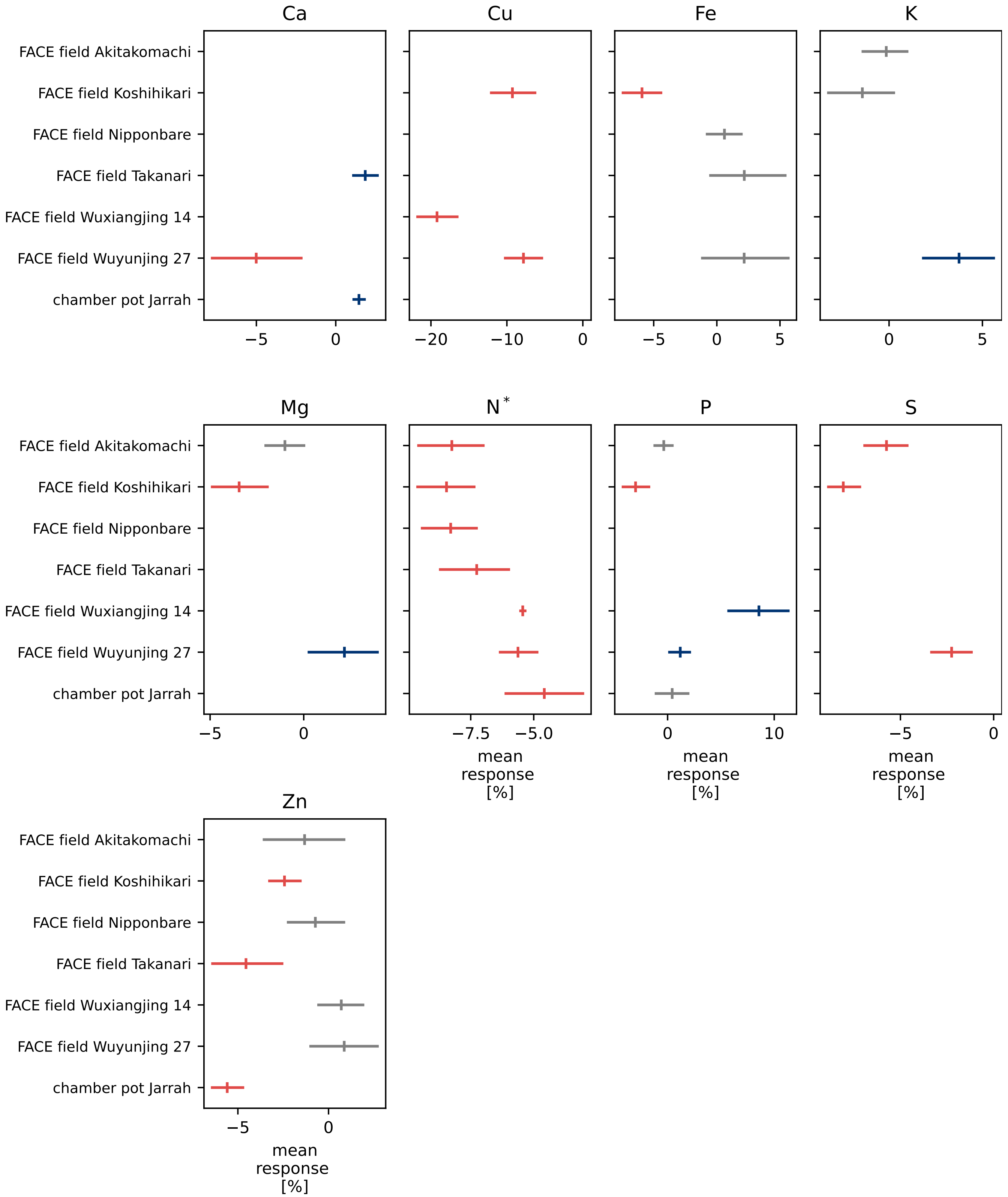

Figure S5. Percent change per element for rice (*Oryza sativa*) cultivars. Cultivars were tested separately per study and plot type. Only high-power results (>0.8) are shown. The arithmetic mean (tick) and the 95% confidence interval (line) are color-coded by statistical significance interpretation and direction of change at $\alpha = 0.05$. N^* means nitrogen proxy and includes protein and nitrogen.

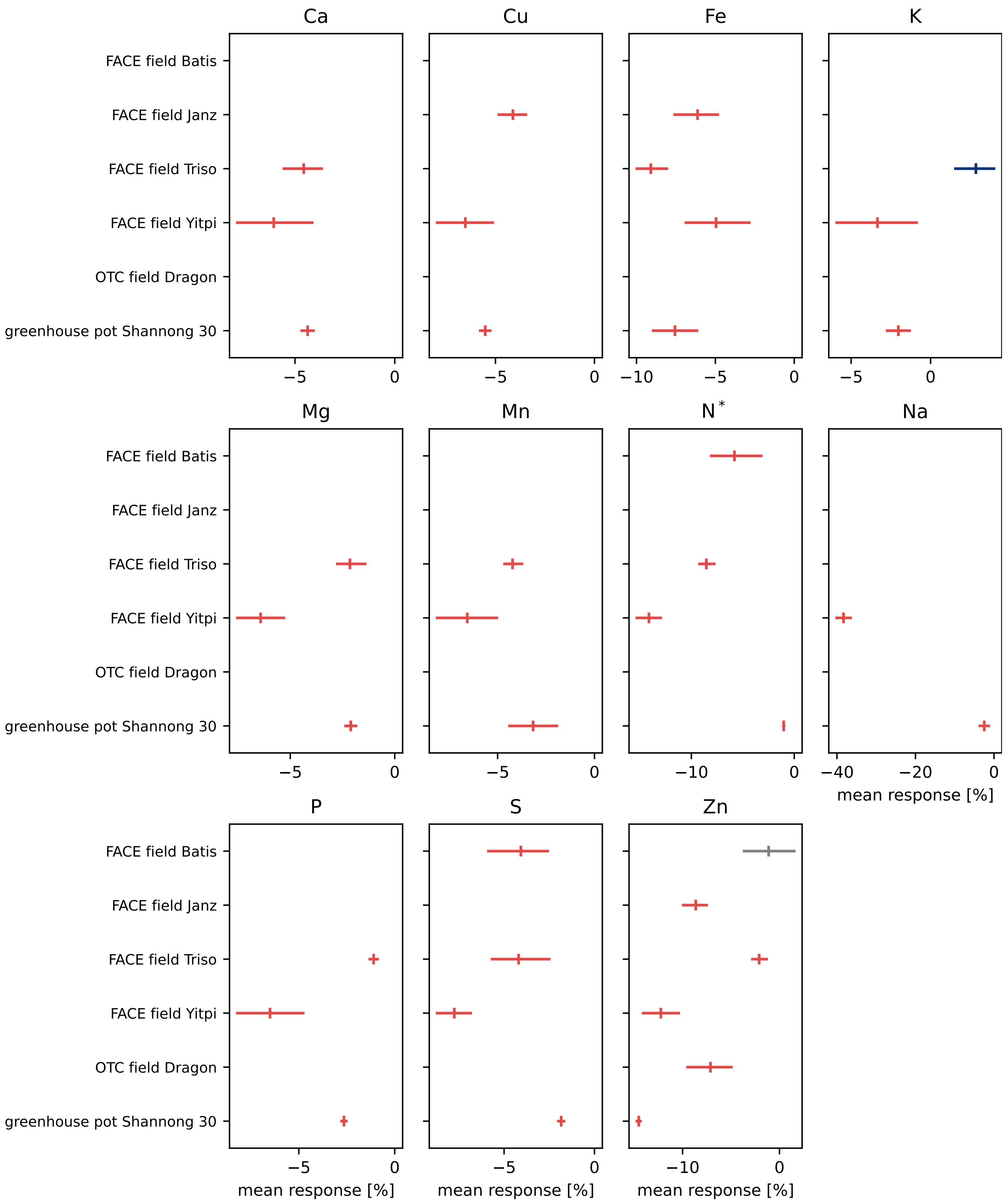

Figure S6. **Percent change per element for wheat (*Triticum aestivum*) cultivars.** Cultivars were tested separately per study and plot type. Only high-power results (>0.8) are shown. The arithmetic mean (tick) and the 95% confidence interval (line) are color-coded by statistical significance interpretation and direction of change at $\alpha = 0.05$. N^* means nitrogen proxy and includes protein and nitrogen.

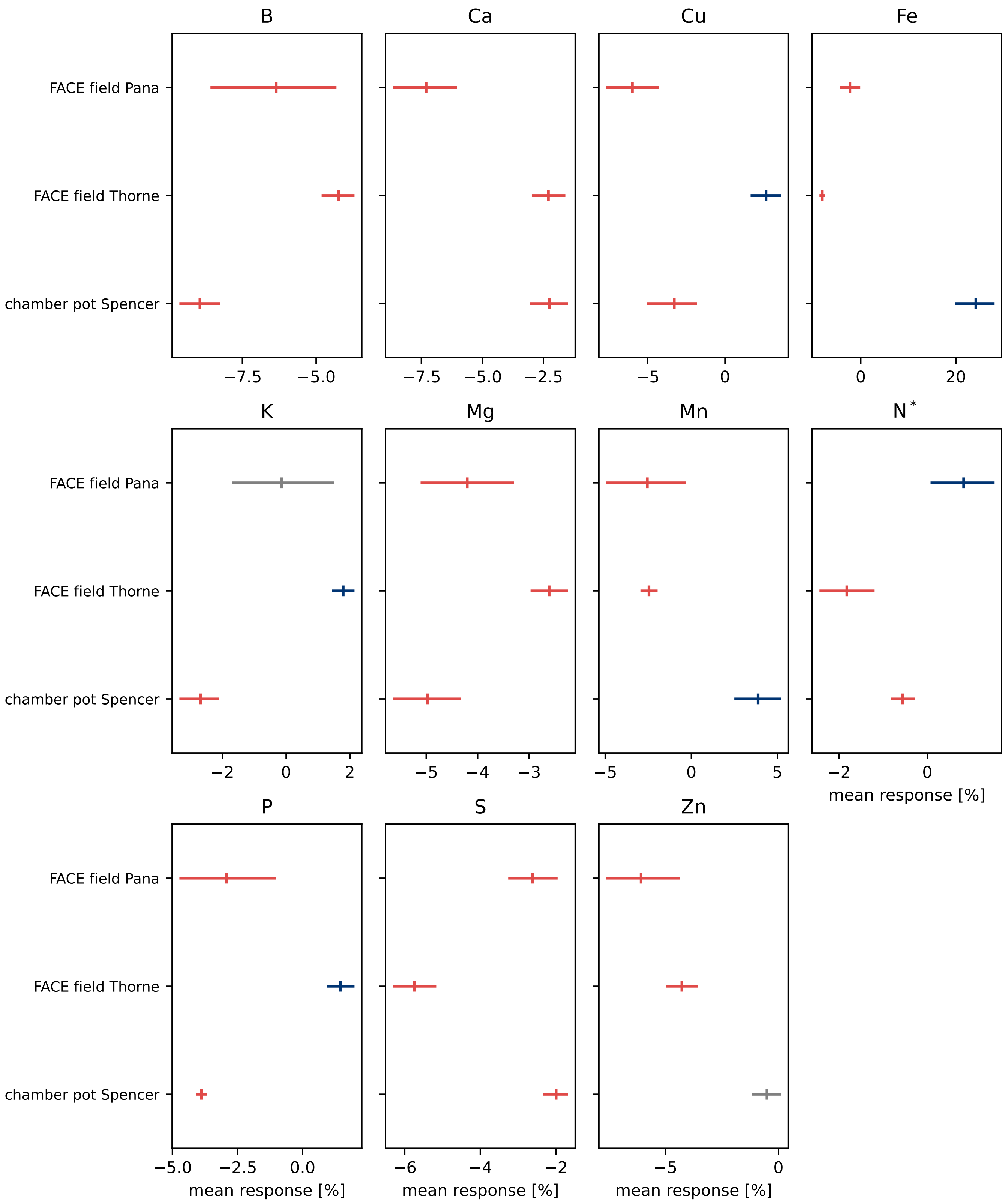

Figure S7. **Percent change per element for soybean (*Glycine max*) cultivars.** Cultivars were tested separately per study and plot type. Only high-power results (>0.8) are shown. The arithmetic mean (tick) and the 95% confidence interval (line) are color-coded by statistical significance interpretation and direction of change at $\alpha = 0.05$. N^* means nitrogen proxy and includes protein and nitrogen.

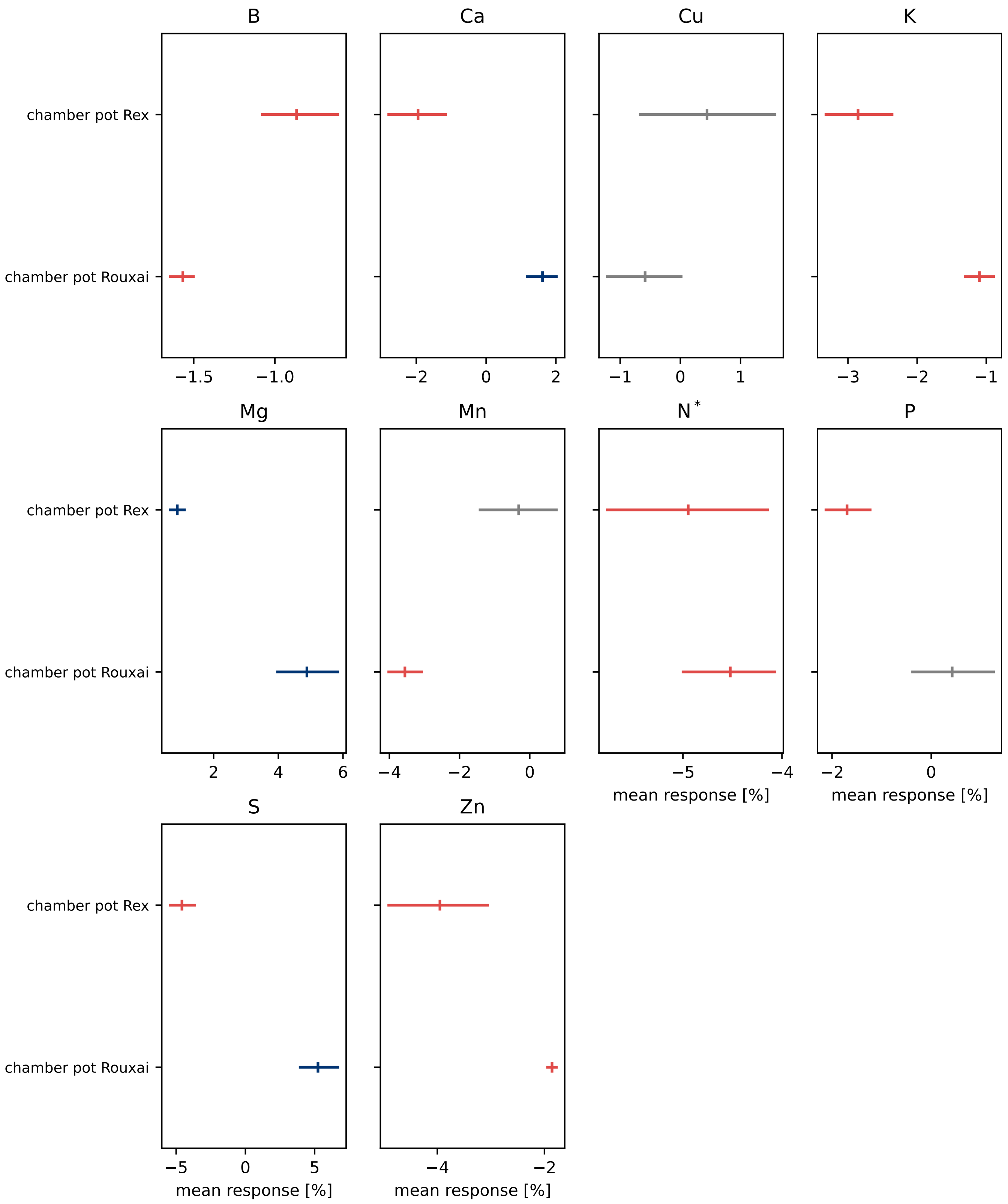

Figure S8. **Percent change per element for lettuce (*Lactuca sativa*) cultivars.** Cultivars were tested separately per study and plot type. Only high-power results (>0.8) are shown. The arithmetic mean (tick) and the 95% confidence interval (line) are color-coded by statistical significance interpretation and direction of change at $\alpha = 0.05$. N^* means nitrogen proxy and includes protein and nitrogen.

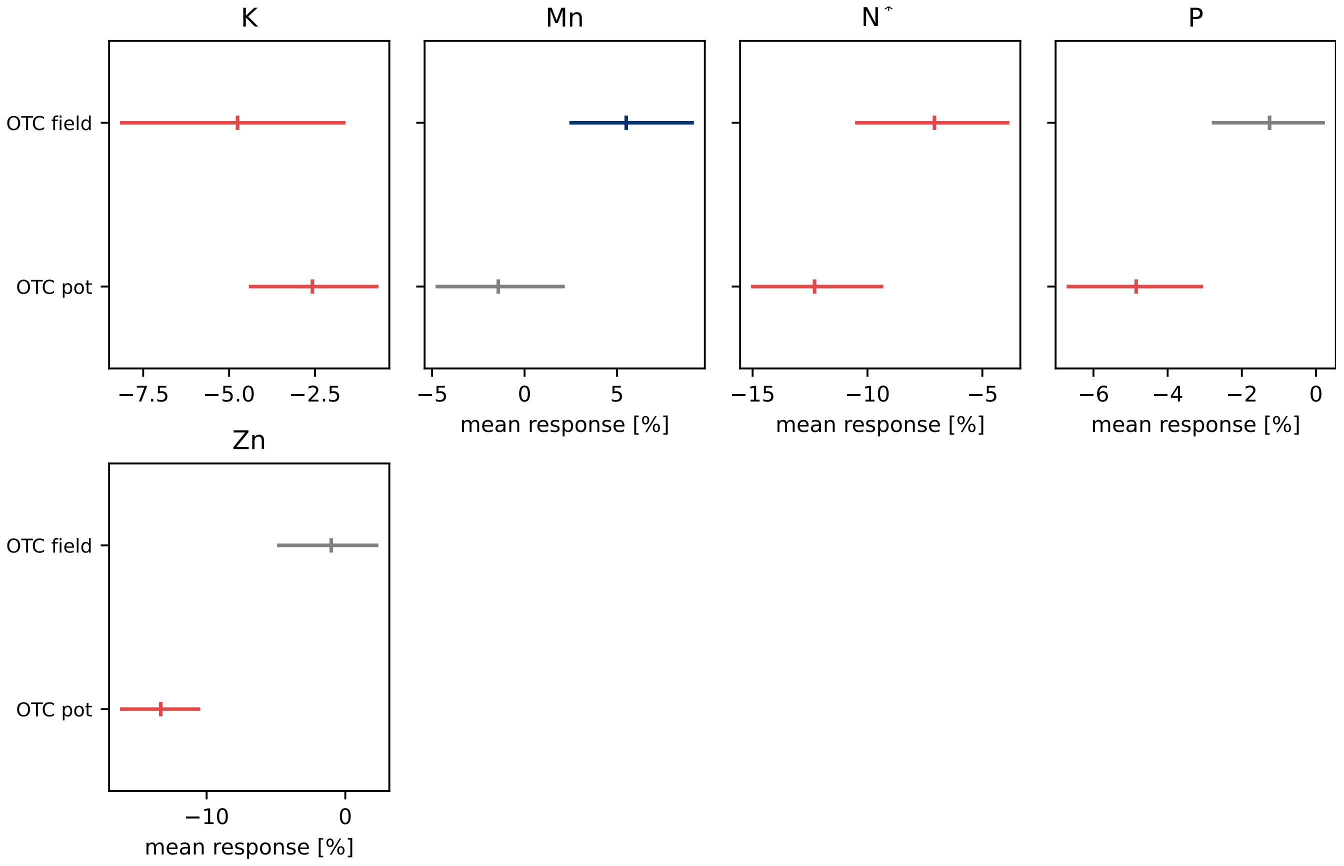

Figure S9. Percent change per element for potato (*Solanum tuberosum*) aggregated over all cultivars. The aggregated cultivars were tested separately per study and plot type. Only high-power results (>0.8) are shown. The arithmetic mean (tick) and the 95% confidence interval (line) are color-coded by statistical significance interpretation and direction of change at α = 0.05. N* means nitrogen proxy and includes protein and nitrogen.
