# Supplementary material for: CO2 Rise Directly Impairs Crop Nutritional Quality
Source: Glob Chang Biol. 2025 Nov 14;31(11):e70568. doi: 10.1111/gcb.70568 (PMC12616468; doi:10.1111/gcb.70568)
Supplement: Supplementary file 2 — Appendix S2: Explanation of changes made between prior databases (Loladze 2014; Myers et al. 2014; Dietterich et al. 2015) and ours for transparency over changes we made and to prevent future users from double‐counting entries. [file GCB-31-e70568-s002.pdf]

CO_2 Rise Directly Impairs Crop Nutritional Quality
Appendix S2: Explanation of Database Discrepancies

By S.F. ter Haar*, P.M. van Bodegom, and L. Scherer
Institute of Environmental Sciences (CML), Leiden University, Leiden, The Netherlands
*email: s.f.ter.haar@cml.leidenuniv.nl

As described in the methods, data was referenced from Loladze (2014) and Dietterich et al. (2015). This process went as follows:

For Dietterich et al. (2015), we took their raw data and reprocessed it for a total of 6,344 pairs. The wheat data was removed because it was covered by a series of papers by Dr. Fernando, leaving a total of 3,432 pairs. The following changes were made to the Dietterich et al. (2015) raw data:

- Changed Ca in Bekoaba (2010, 160) to 0.625 assuming <0.01=0.005
- Changed Ca in Akitakomachi Ca (2010, 80) to 1.9 assuming <0.01=0.005
- Fixed missing B by setting <1 to 0 in program
- Wrote the full names for cultivars (e.g. Glycine max 'Williams' changed to 'Williams 82', 'SY63' to 'Shan You 63', etc)

For Loladze (2014), we filtered the entries to match our inclusion criteria, reprocessed and checked the values, and added in extra (meta)data which consisted mostly of small value adjustments, fixing errant metadata, and/or including protein and phytate.

The ‘source’ column in our database refers to who used the paper in their dataset, and while for ‘Loladze’ does include numbers taken from their work, each number was checked, and may not always match. One would need to be careful in merging the non-edible part of the Loladze (2014) database with ours, as some were reclassified to be edible in our work, while other data was removed. The following table explains the notable changes between the ones used in our work with their original entries in their database.

For Myers et al. (2014), which is based off of Dietterich et al. (2015) database we added in the sources used in their extended databases in a similar process.

Reference	# of Entries		Changes	Notes
	Ours	Loladze (2014)		
Azam et al. 2013	42	39	added in protein per crop	
Barnes & Pffirrman 1992	14	0	added in edible portion of radish, Loladze had the leaves (F, although also edible)	
Baslam et al. 2012	20	0	reclassified as edible	
Carlisle et al. 2012	22	22	no change	
Chagvardieff et al. 1994	14	0	fixed Ca values; listed results as edible	
de la Puente et al. 2000	28	28	no change	
Erbs et al. 2010	72	64	added in N50/N100 and year, added in protein, fixed iron and zinc calculations for Barley, added in cultivar name	
Fangmeier et al. 2002	16	16	No change	
Fangmeier et al. 1997	36	18	added in NF+/NF results	
Fangmeier et al. 1999	8	9	removed Cu because it couldn't be located in the original manuscript	
Fernando et al. 2012a	18	8	Split the data entries by TOS; added in protein; recalculated all values	
Fernando et al. 2012b	4	6	Couldn't locate the same minerals in the manuscript (possibly from Fernando et al. 2012c); recalculated all values	
Fernando et al. 2012c	14	2	Split up protein by TOS, added in Phytate, K, P, Mg, Mn, Na, Cu; recalculated all values	
Fernando et al. 2014	22	16	2014b in our dataset; added in protein; recalculated all values	
Guo et al. 2013	8	8	incorrectly labelled as Guo et al. 2011 (correct in paper, incorrect in database)	
Heagle et al. 2003	120	60	fixed Zn for 80 O3, N for 45 O3 in Superior cultivar, added in 540/370 dataset	
Hogy & Fangmeier 2009	12	11	one B value is actually of Al, added in N	
Hogy et al. 2009	17	16	added in protein per crop	
Hogy et al. 2010	19	18	fixed Ni (inverted sign), added in protein	
Hogy et al. 2013	21	20	one B value is actually of Al, added in N	
Jain et al. 2007	10	0	reclassified as edible	
Khan et al. 2012	28	28	fixed Zn (Astra and Eureka cultivars were switched)	

Li et al. 2010	12	12		had impossible GPS coordinates
Lieffering et al. 2004	22	22	No change	
Manderscheid et al. 1995	39	54	couldn't replicate the numbers from Loladze database, so redid all calculations	
McKeehen et al. 1996	11	0	reclassified lettuce and radish as edible, radish was incorrectly listed as foliage instead of as a root	
Pang et al. 2005	0	18	removed from database due to inclusion criteria (written in English)	
Piikki et al. 2007	10	10	No change	
Pleijel & Danielsson 2009	4	3	added in intermediate CO_2 level in 1995	
Prior et al. 2008	54	0	added in the edible portion from the article	
Seneweera & Conroy 1997	30	30	No change	
Singh et al. 2013	24	10	added in protein, added in experiments where ozone was added to both aCO_2 and eCO_2	
Wroblewitz et al. 2013	48	40	fixed sodium for N50 2002, minute change to Zn N50 2000, added in protein	
Wu et al. 2004	5	4	added in protein	
Yamakawa et al. 2004	6	0	reclassified as edible	
Yang et al. 2007	12	9	added in nitrogen, fixed minor errors	
Ziska et al. 1997	6	2	added in protein, added in results for intermediate CO_2 level	
