# Supplementary material for: CO2 Rise Directly Impairs Crop Nutritional Quality
Source: Glob Chang Biol. 2025 Nov 14;31(11):e70568. doi: 10.1111/gcb.70568 (PMC12616468; doi:10.1111/gcb.70568)
Supplement: Supplementary file 3 — Data S1: The full citation of all the data sources used in the input database. [file GCB-31-e70568-s001.pdf]

By S.F. ter Haar*, P.M. van Bodegom, and L. Scherer
Institute of Environmental Sciences (CML), Leiden University, Leiden, The Netherlands
*email: s.f.ter.haar@cml.leidenuniv.nl

The 'reference' column is the name as used in the input database.

Reference	Full Citation
**A et al 2022**	A, S., Sathee, L., Singh, D., Jha, S. K., Chinnusamy, V., & Singh, M. P. (2022). Interactive effect of elevated CO2 and nitrogen dose reprograms grain ionome and associated gene expression in bread wheat. Plant Physiology and Biochemistry, 179, 134–143. https://doi.org/10.1016/j.plaphy.2022.03.017
**Almuhayawi et al 2021**	Almuhayawi, M. S., Hassan, A. H. A., Al Jaouni, S. K., Alkhalifah, D. H. M., Hozzein, W. N., Selim, S., AbdElgawad, H., & Khamis, G. (2021). Influence of elevated CO2 on nutritive value and health-promoting prospective of three genotypes of Alfalfa sprouts (Medicago Sativa). Food Chemistry, 340, 128147. https://doi.org/10.1016/j.foodchem.2020.128147
**Asif et al 2017**	Asif, M., Yilmaz, O., & Ozturk, L. (2017). Elevated carbon dioxide ameliorates the effect of Zn deficiency and terminal drought on wheat grain yield but compromises nutritional quality. Plant and Soil, 411(1), 57–67. https://doi.org/10.1007/s11104-016-2996-9
**Asif et al 2018**	Asif, M., Tunc, C. E., & Ozturk, L. (2018). Changes in yield attributes and K allocation in wheat as affected by K deficiency and elevated CO2. Plant and Soil, 426(1), 153–162. https://doi.org/10.1007/s11104-018-3603-z
**Azam et al 2013**	Azam, A., Khan, I., Mahmood, A., & Hameed, A. (2013). Yield, chemical composition and nutritional quality responses of carrot, radish and turnip to elevated atmospheric carbon dioxide. Journal of the Science of Food and Agriculture, 93(13), 3237–3244. https://doi.org/10.1002/jsfa.6165
**Bagudam et al 2023**	Bagudam, R., Kancherla, E., Abady, S., Wankhade, A. P., Deshmukh, D. B., Vemula, A., Kadirimangalam, S. R., Kumar, S. S., Reddy, S. N., & Pasupuleti, J. (2023). Influence of elevated CO2 on growth, yield, haulm, and kernel quality of groundnut (Arachis hypogaea L.). Acta Physiologiae Plantarum, 45(5), 67. https://doi.org/10.1007/s11738-023-03553-4
**Balbinot et al 2021**	Balbinot, A., da Rosa Feijó, A., Fipke, M. V., Rockenbach, D., Massey, J. H., Camargo, E. R., Mesko, M. F., Scaglioni, P. T., & de Avila, L. A. (2021). Effects of Elevated Atmospheric CO2 Concentration and Water Regime on Rice Yield, Water Use Efficiency, and Arsenic and Cadmium Accumulation in Grain. Agriculture, 11(8), 705. https://doi.org/10.3390/agriculture11080705
**Barnes & Pffirrman 1992**	Barnes, J. D., & Pfirrmann, T. (1992). The influence of CO2 and O3, singly and in combination, on gas exchange, growth and nutrient status of radish (Raphanus sativus L.). New Phytologist, 121(3), 403–412. https://doi.org/10.1111/j.1469-8137.1992.tb02940.x
**Baslam et al 2012**	Baslam, M., Garmendia, I., & Goicoechea, N. (2012). Elevated CO2 may impair the beneficial effect of arbuscular mycorrhizal fungi on the mineral and phytochemical quality of lettuce. Annals of Applied Biology, 161(2), 180–191. https://doi.org/10.1111/j.1744-7348.2012.00563.x
**Behboudian and Tod 1995**	Behboudian, M. H., & Tod, C. (1995). Postharvest Attributes of `Virosa' Tomato Fruit Produced in an Enriched Carbon Dioxide Environment. HortScience, 30(3), 490–491. https://doi.org/10.21273/HORTSCI.30.3.490
**Beleggia et al 2018**	Beleggia, R., Fragasso, M., Miglietta, F., Cattivelli, L., Menga, V., Nigro, F., Pecchioni, N., & Fares, C. (2018). Mineral composition of durum wheat grain and pasta under increasing atmospheric CO2 concentrations. Food Chemistry, 242, 53–61. https://doi.org/10.1016/j.foodchem.2017.09.012

**Bellaloui et al 2016**	Bellaloui, N., Hu, Y., Mengistu, A., Abbas, H. K., Kassem, M. A., & Tigabu, M. (2016). Elevated Atmospheric Carbon Dioxide and Temperature Affect Seed Composition, Mineral Nutrition, and 15N and 13C Dynamics in Soybean Genotypes under Controlled Environments. Atlas Journal of Plant Biology, 56–65. https://doi.org/10.5147/ajpb.v0i0.114
**Boufeldja et al 2022**	Boufeldja, L., Brandt, D., Guzman, C., Vitou, M., Boudard, F., Morel, S., Servent, A., Dhuique-Mayer, C., Ollier, L., Duchamp, O., Portet, K., Dubos, C., & Poucheret, P. (2022). Effect of Elevated Carbon Dioxide Exposure on Nutrition-Health Properties of Micro-Tom Tomatoes. Molecules, 27(11), 3592. https://doi.org/10.3390/molecules27113592
**Boufeldja et al 2023**	Boufeldja, L., Boudard, F., Portet, K., Guzman, C., Morel, S., Berger, N., Duchamp, O., Dhuique-Mayer, C., Dubos, C., & Poucheret, P. (2023). The Impact of Elevated Atmospheric Carbon Dioxide Exposure on Magic Tomatoes’ Nutrition-Health Properties. International Journal of Molecular Sciences, 24(16), 12815. https://doi.org/10.3390/ijms241612815
**Carlisle et al 2012**	Carlisle, E., Myers, S., Raboy, V., & Bloom, A. (2012). The Effects of Inorganic Nitrogen form and CO2 Concentration on Wheat Yield and Nutrient Accumulation and Distribution. Frontiers in Plant Science, 3. https://www.frontiersin.org/articles/10.3389/fpls.2012.00195
**Chagvardieff et al 1994**	Chagvardieff, P., d’Aletto, T., & André, M. (1994). Specific effects of irradiance and CO2 concentration doublings on productivity and mineral content in lettuce. Advances in Space Research, 14(11), 269–275. https://doi.org/10.1016/0273-1177(94)90307-7
**Chaturvedi et al 2017**	Chaturvedi, A. K., Bahuguna, R. N., Pal, M., Shah, D., Maurya, S., & Jagadish, K. S. V. (2017). Elevated CO2 and heat stress interactions affect grain yield, quality and mineral nutrient composition in rice under field conditions. Field Crops Research, 206, 149–157. https://doi.org/10.1016/j.fcr.2017.02.018
**de la Puente et al 2000**	de La Puente, P Pérez, Rafael Martínez-Carrasco, Rosa Morcuende, & I.M. Molino. (2000). Action of elevated CO2 and high temperatures on the mineral chemical composition of two varieties of wheat. *Agrochimica*, *44*, 221–230.
**Deuchande and Vasconcelos 2023**	Deuchande, T., & Vasconcelos, M. (2023). Combined effect of elevated CO2 and Fe deficiency on common bean metabolism and mineral profile. Plant and Soil, 496, 139–160. https://doi.org/10.1007/s11104-023-06010-w
**Dier et al 2020**	Dier, M., Hüther, L., Schulze, W. X., Erbs, M., Köhler, P., Weigel, H.-J., Manderscheid, R., & Zörb, C. (2020). Elevated Atmospheric CO2 Concentration Has Limited Effect on Wheat Grain Quality Regardless of Nitrogen Supply. Journal of Agricultural and Food Chemistry, 68(12), 3711–3721. https://doi.org/10.1021/acs.jafc.9b07817
**Dietterich et al 2015**	Dietterich, L. H., Zanobetti, A., Kloog, I., Huybers, P., Leakey, A. D. B., Bloom, A. J., Carlisle, E., Fernando, N., Fitzgerald, G., Hasegawa, T., Holbrook, N. M., Nelson, R. L., Norton, R., Ottman, M. J., Raboy, V., Sakai, H., Sartor, K. A., Schwartz, J., Seneweera, S., … Myers, S. S. (2015). Impacts of elevated atmospheric CO2 on nutrient content of important food crops. Scientific Data, 2(1), 150036. https://doi.org/10.1038/sdata.2015.36
**Dong et al 2018a**	Dong, J., Li, X., Nazim, G., & Duan, Z. (2018). Interactive effects of elevated carbon dioxide and nitrogen availability on fruit quality of cucumber (Cucumis sativus L.). Journal of Integrative Agriculture, 17(11), 2438–2446. https://doi.org/10.1016/S2095-3119(18)62005-2
**Dong et al 2018b**	Dong, J., Xu, Q., Gruda, N., Chu, W., Li, X., & Duan, Z. (2018). Elevated and super-elevated CO2 differ in their interactive effects with nitrogen availability on fruit yield and quality of cucumber. Journal of the Science of Food and Agriculture, 98(12), 4509–4516. https://doi.org/10.1002/jsfa.8976

**Du et al 2017**	Du, W., Gardea-Torresdey, J. L., Xie, Y., Yin, Y., Zhu, J., Zhang, X., Ji, R., Gu, K., Peralta-Videa, J. R., & Guo, H. (2017). Elevated CO2 levels modify TiO2 nanoparticle effects on rice and soil microbial communities. Science of The Total Environment, 578, 408–416. https://doi.org/10.1016/j.scitotenv.2016.10.197
**Erbs et 2010**	Erbs, M., Manderscheid, R., Jansen, G., Seddig, S., Pacholski, A., & Weigel, H.-J. (2010). Effects of free-air CO2 enrichment and nitrogen supply on grain quality parameters and elemental composition of wheat and barley grown in a crop rotation. Agriculture, Ecosystems & Environment, 136(1), 59–68. https://doi.org/10.1016/j.agee.2009.11.009
**Fangmeier et al 1997**	Fangmeier, A., Grüters, U., Högy, P., Vermehren, B., & Jäger, H.-J. (1997). Effects of elevated CO2, nitrogen supply and tropospheric ozone on spring wheat—II. Nutrients (N, P, K, S, Ca, Mg, Fe, Mn, Zn). Environmental Pollution, 96(1), 43–59. https://doi.org/10.1016/S0269-7491(97)00013-4
**Fangmeier et al 1999**	Fangmeier, A., De Temmerman, L., Mortensen, L., Kemp, K., Burke, J., Mitchell, R., van Oijen, M., & Weigel, H.-J. (1999). Effects on nutrients and on grain quality in spring wheat crops grown under elevated CO2 concentrations and stress conditions in the European, multiple-site experiment 'ESPACE-wheat.' European Journal of Agronomy, 10(3), 215–229. https://doi.org/10.1016/S1161-0301(99)00012-X
**Fangmeier et al 2002**	Fangmeier, A., De Temmerman, L., Black, C., Persson, K., & Vorne, V. (2002). Effects of elevated CO2 and/or ozone on nutrient concentrations and nutrient uptake of potatoes. European Journal of Agronomy, 17(4), 353–368. https://doi.org/10.1016/S1161-0301(02)00071-0
**Fernando et al 2012a**	Fernando, N., Panozzo, J., Tausz, M., Norton, R., Fitzgerald, G., & Seneweera, S. (2012). Rising atmospheric CO2 concentration affects mineral nutrient and protein concentration of wheat grain. Food Chemistry, 133(4), 1307–1311. https://doi.org/10.1016/j.foodchem.2012.01.105
**Fernando et al 2012b**	Fernando, N., Panozzo, J., Tausz, M., Norton, R. M., Fitzgerald, G., & Seneweera, S. (2012). Elevated Atmospheric CO2 Affects Grain Sulfur Concentration and Grain Nitrogen/Sulfur Ratio of Wheat (Triticum aestivum L.). In L. J. De Kok, L. Tabe, M. Tausz, M. J. Hawkesford, R. Hoefgen, M. T. McManus, R. M. Norton, H. Rennenberg, K. Saito, & E. Schnug (Eds.), Sulfur Metabolism in Plants (pp. 231–236). Springer Netherlands. https://doi.org/10.1007/978-94-007-4450-9_28
**Fernando et al 2012c**	Fernando, N., Panozzo, J., Tausz, M., Norton, R. M., Fitzgerald, G. J., Myers, S., Walker, C., Stangoulis, J., & Seneweera, S. (2012). Wheat grain quality under increasing atmospheric CO2 concentrations in a semi-arid cropping system. Journal of Cereal Science, 56(3), 684–690. https://doi.org/10.1016/j.jcs.2012.07.010
**Fernando et al 2014a**	Fernando, N., Panozzo, J., Tausz, M., Norton, R. M., Fitzgerald, G. J., Myers, S., Nicolas, M. E., & Seneweera, S. (2014). Intra-specific variation of wheat grain quality in response to elevated [CO2] at two sowing times under rain-fed and irrigation treatments. Journal of Cereal Science, 59(2), 137–144. https://doi.org/10.1016/j.jcs.2013.12.002
**Fernando et al 2014b**	Fernando, N., Panozzo, J., Tausz, M., Norton, R. M., Neumann, N., Fitzgerald, G. J., & Seneweera, S. (2014). Elevated CO2 alters grain quality of two bread wheat cultivars grown under different environmental conditions. Agriculture, Ecosystems & Environment, 185, 24–33. https://doi.org/10.1016/j.agee.2013.11.023
**Gao et al 2021**	Gao, B., Hu, S., Jing, L., Wang, Y., Zhu, J., Wang, K., Li, H., Sun, X., Wang, Y., & Yang, L. (2021). Impact of Elevated CO2 and Reducing the Source-Sink Ratio by Partial Defoliation on Rice Grain Quality – A 3-Year Free-Air CO2 Enrichment Study. Frontiers in Plant Science, 12, 788104. https://doi.org/10.3389/fpls.2021.788104

**Garmendia et al 2022**	Garmendia, I., Rashidi, S., Quezada-Salirrosas, M. R., & Goicoechea, N. (2022). Atmospheric CO2 concentration affects the life cycle, yield, and fruit quality of early maturing edible legume cultivars. Journal of the Science of Food and Agriculture, 102(10), 3964–3971. https://doi.org/10.1002/jsfa.11743
**Giri et al 2016**	Giri, A., Armstrong, B., & Rajashekar, C. B. (2016). Elevated Carbon Dioxide Level Suppresses Nutritional Quality of Lettuce and Spinach. American Journal of Plant Sciences, 7(1), 246–258. https://doi.org/10.4236/ajps.2016.71024
**Goicoechea et al 2016**	Goicoechea, N., Bettoni, M. M., Fuertes-Mendizábal, T., González-Murua, C., & Aranjuelo, I. (2016). Durum wheat quality traits affected by mycorrhizal inoculation, water availability and atmospheric CO2 concentration. Crop and Pasture Science, 67(2), 147–155. https://doi.org/10.1071/CP15212
**Gong et al 2021**	Gong, Z., Dong, L., Lam, S., Zhang, D., Zong, Y., Hao, X., & Li, P. (2021). Nutritional quality in response to elevated CO2 concentration in foxtail millet (Setaria italica). Journal of Cereal Science, 102, 103318. https://doi.org/10.1016/j.jcs.2021.103318
**Goufo et al 2014**	Goufo, P., Falco, V., Brites, C., Wessel, D. F., Kratz, S., Rosa, E. A. S., Carranca, C., & Trindade, H. (2014). Effect of Elevated Carbon Dioxide Concentration on Rice Quality: Nutritive Value, Color, Milling, Cooking, and Eating Qualities. Cereal Chemistry, 91(5), 513–521. https://doi.org/10.1094/CCHEM-12-13-0256-R
**Guo et al 2011**	Guo, H., Zhu, J., Zhou, H., Sun, Y., Yin, Y., Pei, D., Ji, R., Wu, J., & Wang, X. (2011). Elevated CO 2 Levels Affects the Concentrations of Copper and Cadmium in Crops Grown in Soil Contaminated with Heavy Metals under Fully Open-Air Field Conditions. Environmental Science & Technology, 45(16), 6997–7003. https://doi.org/10.1021/es2001584
**Guo et al 2022**	Guo, X., Huang, B., Zhang, H., Cai, C., Li, G., Li, H., Zhang, Y., Struik, P. C., Liu, Z., Dong, M., Ni, R., Pan, G., Liu, X., Chen, W., Luo, W., & Yin, X. (2022). T-FACE studies reveal that increased temperature exerts an effect opposite to that of elevated CO2 on nutrient concentration and bioavailability in rice and wheat grains. Food and Energy Security, 11(1), e336. https://doi.org/10.1002/fes3.336
**Heagle et al 2003**	Heagle, A. S., Miller, J. E., & Pursley, W. A. (2003). Growth and Yield Responses of Potato to Mixtures of Carbon Dioxide and Ozone. Journal of Environmental Quality, 32(5), 1603–1610. https://doi.org/10.2134/jeq2003.1603
**Hogy & Fangmeier 2009**	Högy, P., & Fangmeier, A. (2009). Atmospheric CO2 enrichment affects potatoes: 2. Tuber quality traits. European Journal of Agronomy, 30(2), 85–94. https://doi.org/10.1016/j.eja.2008.07.006
**Hogy et al 2009a**	Högy, P., Wieser, H., Köhler, P., Schwadorf, K., Breuer, J., Franzaring, J., Muntifering, R., & Fangmeier, A. (2009). Effects of elevated CO2 on grain yield and quality of wheat: Results from a 3-year free-air CO2 enrichment experiment. Plant Biology, 11(s1), 60–69. https://doi.org/10.1111/j.1438-8677.2009.00230.x
**Hogy et al 2009b**	Högy, P., Wieser, H., Köhler, P., Schwadorf, K., Breuer, J., Erbs, M., Weber, S., & Fangmeier, A. (2009). Does elevated atmospheric CO2 allow for sufficient wheat grain quality in the future? *Journal of Applied Botany and Food Quality*, *82*, 114–121.
**Hogy et al 2010**	Högy, P., Franzaring, J., Schwadorf, K., Breuer, J., Schütze, W., & Fangmeier, A. (2010). Effects of free-air CO2 enrichment on energy traits and seed quality of oilseed rape. Agriculture, Ecosystems & Environment, 139(1), 239–244. https://doi.org/10.1016/j.agee.2010.08.009
**Hogy et al 2013**	Högy, P., Brunnbauer, M., Koehler, P., Schwadorf, K., Breuer, J., Franzaring, J., Zhunusbayeva, D., & Fangmeier, A. (2013). Grain quality characteristics of spring wheat (Triticum aestivum) as affected by free-air CO2 enrichment. Environmental and Experimental Botany, 88, 11–18. https://doi.org/10.1016/j.envexpbot.2011.12.007

**Holley et al 2022**	Holley, J., Mattson, N., Ashenafi, E., & Nyman, M. (2022). The Impact of CO2 Enrichment on Biomass, Carotenoids, Xanthophyll, and Mineral Content of Lettuce (Lactuca sativa L.). Horticulturae, 8(9), 820. https://doi.org/10.3390/horticulturae8090820
**Jain 2007**	Jain, V., Pal, M., Raj, A., & Khetarpal, S. (2007). Photosynthesis and nutrient composition of spinach and fenugreek grown under elevated carbon dioxide concentration. Biologia Plantarum, 51(3), 559–562. https://doi.org/10.1007/s10535-007-0122-9
**Jena et al 2018**	Jena, U. R., Swain, D. K., Hazra, K. K., & Maiti, M. K. (2018). Effect of elevated [CO2] on yield, intra-plant nutrient dynamics, and grain quality of rice cultivars in eastern India. Journal of the Science of Food and Agriculture, 98(15), 5841–5852. https://doi.org/10.1002/jsfa.9135
**Jin et al 2019**	Jin, J., Armstrong, R., & Tang, C. (2019). Impact of elevated CO2 on grain nutrient concentration varies with crops and soils – A long-term FACE study. Science of The Total Environment, 651, 2641–2647. https://doi.org/10.1016/j.scitotenv.2018.10.170
**Khan et al 2013**	Khan, I., Azam, A., & Mahmood, A. (2013). The impact of enhanced atmospheric carbon dioxide on yield, proximate composition, elemental concentration, fatty acid and vitamin C contents of tomato (Lycopersicon esculentum). Environmental Monitoring and Assessment, 185(1), 205–214. https://doi.org/10.1007/s10661-012-2544-x
**Kohler et al 2019**	Köhler, I. H., Huber, S. C., Bernacchi, C. J., & Baxter, I. R. (2019). Increased temperatures may safeguard the nutritional quality of crops under future elevated CO2 concentrations. The Plant Journal, 97(5), 872–886. https://doi.org/10.1111/tpj.14166
**Kumari and Agrawal 2014**	Kumari, S., & Agrawal, M. (2014). Growth, yield and quality attributes of a tropical potato variety (Solanum tuberosum L. cv Kufri chandramukhi) under ambient and elevated carbon dioxide and ozone and their interactions. Ecotoxicology and Environmental Safety, 101, 146–156. https://doi.org/10.1016/j.ecoenv.2013.12.021
**La et al 2009**	La, G., Fang, P., Teng, Y., Li, Y., & Lin, X. (2009). Effect of CO2 enrichment on the glucosinolate contents under different nitrogen levels in bolting stem of Chinese kale (Brassica alboglabra L.). Journal of Zhejiang University SCIENCE B, 10(6), 454–464. https://doi.org/10.1631/jzus.B0820354
**Li et al 2010**	Li, Z., Tang, S., Deng, X., Wang, R., & Song, Z. (2010). Contrasting effects of elevated CO2 on Cu and Cd uptake by different rice varieties grown on contaminated soils with two levels of metals: Implication for phytoextraction and food safety. Journal of Hazardous Materials, 177(1), 352–361. https://doi.org/10.1016/j.jhazmat.2009.12.039
**Li et al 2016**	Li, X., Jiang, D., & Liu, F. (2016). Soil warming enhances the hidden shift of elemental stoichiometry by elevated CO2 in wheat. Scientific Reports, 6(1), 23313. https://doi.org/10.1038/srep23313
**Li et al 2018**	Li, Y., Yu, Z., Jin, J., Zhang, Q., Wang, G., Liu, C., Wu, J., Wang, C., & Liu, X. (2018). Impact of Elevated CO2 on Seed Quality of Soybean at the Fresh Edible and Mature Stages. Frontiers in Plant Science, 9, 1413. https://doi.org/10.3389/fpls.2018.01413
**Li et al 2019**	Li, X., Ulfat, A., Lv, Z., Fang, L., Jiang, D., & Liu, F. (2019). Effect of multigenerational exposure to elevated atmospheric CO2 concentration on grain quality in wheat. Environmental and Experimental Botany, 157, 310–319. https://doi.org/10.1016/j.envexpbot.2018.10.028
**Lieffering et al 2004**	Lieffering, M., Kim, H.-Y., Kobayashi, K., & Okada, M. (2004). The impact of elevated CO2 on the elemental concentrations of field-grown rice grains. Field Crops Research, 88(2), 279–286. https://doi.org/10.1016/j.fcr.2004.01.004
**Luo et al 2019**	Luo, X.-S., Zhang, D., Hu, Z., Liu, C., Zhao, Z., Sun, W., Fang, X., & Fan, P. (2019). Effects of elevated carbon dioxide on metal transport in soil-crop system: Results from a field rice and wheat experiment. Journal of Soils and Sediments, 19(11), 3742–3748. https://doi.org/10.1007/s11368-019-02329-z

**Ma et al 2007**	Ma, H., Zhu, J., Xie, Z., Liu, G., Zeng, Q., & Han, Y. (2007). Responses of rice and winter wheat to free-air CO2 enrichment (China FACE) at rice/wheat rotation system. Plant and Soil, 294(1), 137–146. https://doi.org/10.1007/s11104-007-9241-5
**Manderscheid 1995**	Manderscheid, R., Bender, J., Jäger, H.-J., & Weigel, H.-J. (1995). Effects of season long CO2 enrichment on cereals. II. Nutrient concentrations and grain quality. Agriculture, Ecosystems & Environment, 54(3), 175–185. https://doi.org/10.1016/0167-8809(95)00602-O
**Manderscheid et al 2010**	Manderscheid, R., Pacholski, A., & Weigel, H.-J. (2010). Effect of free air carbon dioxide enrichment combined with two nitrogen levels on growth, yield and yield quality of sugar beet: Evidence for a sink limitation of beet growth under elevated CO2. European Journal of Agronomy, 32(3), 228–239. https://doi.org/10.1016/j.eja.2009.12.002
**McKeehen et al 1996**	McKeehen, J. D., Smart, D. J., Mackowiak, C. L., Wheeler, R. M., & Nielsen, S. S. (1996). Effect of CO2 levels on nutrient content of lettuce and radish. Advances in Space Research, 18(4), 85–92. https://doi.org/10.1016/0273-1177(95)00864-B
**Nakandalage et al 2023**	Nakandalage, N., Milham, P. J., Holford, P., & Seneweera, S. (2023). Luxury Zinc Supply Prevents the Depression of Grain Nitrogen Concentrations in Rice (Oryza sativa L.) Typically Induced by Elevated CO2. Plants (Basel, Switzerland), 12(4), 839. https://doi.org/10.3390/plants12040839
**Parvin et al 2019**	Parvin, S., Uddin, S., Tausz-Posch, S., Armstrong, R., Fitzgerald, G., & Tausz, M. (2019). Grain mineral quality of dryland legumes as affected by elevated CO2 and drought: A FACE study on lentil (Lens culinaris) and faba bean (Vicia faba). Crop and Pasture Science, 70(3), 244–253. https://doi.org/10.1071/CP18421
**Perez-Lopez et al 2015a**	Pérez-López, U., Miranda-Apodaca, J., Lacuesta, M., Mena-Petite, A., & Muñoz-Rueda, A. (2015). Growth and nutritional quality improvement in two differently pigmented lettuce cultivars grown under elevated CO2 and/or salinity. Scientia Horticulturae, 195, 56–66. https://doi.org/10.1016/j.scienta.2015.08.034
**Perez-Lopez et al 2015b**	Pérez-López, U., Miranda-Apodaca, J., Muñoz-Rueda, A., & Mena-Petite, A. (2015). Interacting effects of high light and elevated CO2 on the nutraceutical quality of two differently pigmented Lactuca sativa cultivars (Blonde of Paris Batavia and Oak Leaf). Scientia Horticulturae, 191, 38–48. https://doi.org/10.1016/j.scienta.2015.04.030
**Piikki et al 2007**	Piikki, K., Vorne, V., Ojanperä, K., & Pleijel, H. (2007). Impact of elevated O3 and CO2 exposure on potato (Solanum tuberosum L. cv. Bintje) tuber macronutrients (N, P, K, Mg, Ca). Agriculture, Ecosystems & Environment, 118(1), 55–64. https://doi.org/10.1016/j.agee.2006.04.012
**Pimenta et al 2023**	Pimenta, T. M., Souza, G. A., Brito, F. A. L., Teixeira, L. S., Arruda, R. S., Henschel, J. M., Zsögön, A., & Ribeiro, D. M. (2023). The impact of elevated CO2 concentration on fruit size, quality, and mineral nutrient composition in tomato varies with temperature regimen during growing season. Plant Growth Regulation, 100(2), 519–530. https://doi.org/10.1007/s10725-022-00889-8
**Pinero et al 2017**	Piñero, M. C., Otálora, G., Porras, M. E., Sánchez-Guerrero, M. C., Lorenzo, P., Medrano, E., & del Amor, F. M. (2017). The Form in Which Nitrogen Is Supplied Affects the Polyamines, Amino Acids, and Mineral Composition of Sweet Pepper Fruit under an Elevated CO2 Concentration. Journal of Agricultural and Food Chemistry, 65(4), 711–717. https://doi.org/10.1021/acs.jafc.6b04118
**Pinero et al 2018**	Piñero, M. C., Pérez-Jiménez, M., López-Marín, J., & del Amor, F. M. (2018). Fruit quality of sweet pepper as affected by foliar Ca applications to mitigate the supply of saline water under a climate change scenario. Journal of the Science of Food and Agriculture, 98(3), 1071–1078. https://doi.org/10.1002/jsfa.8557

**Pleijel & Danielsson 2009**	Pleijel, H., & Danielsson, H. (2009). Yield dilution of grain Zn in wheat grown in open-top chamber experiments with elevated CO2 and O3 exposure. Journal of Cereal Science, 50(2), 278–282. https://doi.org/10.1016/j.jcs.2009.06.009
**Prior et al 2008**	Prior, S. A., Runion, G. B., Rogers, H. H., & Torbert, H. A. (2008). Effects of Atmospheric CO2 Enrichment on Crop Nutrient Dynamics under No-Till Conditions. Journal of Plant Nutrition, 31(4), 758–773. https://doi.org/10.1080/01904160801928364
**Raj and Sanyal 2017**	Raj, A., & Sanyal, A. (2017). Impact of elevated carbon dioxide and different nitrogen doses on grain quality of rice (Oryza sativa L.). Chemical Science Review and Letters, 6(23), 1923–1930. https://doi.org/10.1002/jsfa.7545
**Raviteja et al 2021**	Raviteja, D. H., Kumar, T., Sharma, S., Kumar, A., Singh, M. P., & Pandey, R. (2021). Impact of elevated CO2 and phosphorus nutrition on mineral composition and cooking quality of rice. Plant Physiology Reports, 26(4), 687–698. https://doi.org/10.1007/s40502-021-00623-y
**Reich et al 2015**	Reich, M., Meerakker, A. N. van den, Parmar, S., Hawkesford, M. J., & Kok, de L. (2015). Temperature determines size and direction of effects of elevated CO2 and nitrogen form on yield quantity and quality of Chinese cabbage. Plant Biology (Stuttgart, Germany), 18(S1), 63–75. https://doi.org/10.1111/plb.12396
**Saha et al 2015**	Saha, S., Chakraborty, D., Sehgal, V. K., & Pal, M. (2015). Potential impact of rising atmospheric CO2 on quality of grains in chickpea (Cicer arietinum L.). Food Chemistry, 187, 431–436. https://doi.org/10.1016/j.foodchem.2015.04.116
**Saleh et al 2018**	Saleh, A. M., Selim, S., Jaouni, S. A., & AbdElgawad, H. (2018). CO2 enrichment can enhance the nutritional and health benefits of parsley (Petroselinum crispum L.) and dill (Anethum graveolens L.). Food Chemistry, 269, 519–526. https://doi.org/10.1016/j.foodchem.2018.07.046
**Schonhof et al 2007**	Schonhof, I., Kläring, H.-P., Krumbein, A., & Schreiner, M. (2007). Interaction Between Atmospheric CO2 and Glucosinolates in Broccoli. Journal of Chemical Ecology, 33(1), 105–114. https://doi.org/10.1007/s10886-006-9202-0
**Segura et al 2001**	Segura, M. L., Parra, J. F., Lorenzo, P., Sánchez-Guerrero, M. C., & Medrano, E. (2001). THE EFFECTS OF CO2 ENRICHMENT ON CUCUMBER GROWTH UNDER GREENHOUSE CONDITIONS. Acta Horticulturae, 559(559), 217–222. https://doi.org/10.17660/ActaHortic.2001.559.31
**Seneweera & Conroy 1997**	Seneweera, S. P., & Conroy, J. P. (1997). Growth, grain yield and quality of rice (Oryza sativa L.) in response to elevated CO 2 and phosphorus nutrition. Soil Science and Plant Nutrition, 43(sup1), 1131–1136. https://doi.org/10.1080/00380768.1997.11863730
**Seneweera et al 1996**	Seneweera, S., Blakeney, A., Milham, P., Basra, A. S., Barlow, E. W. R., & Conroy', J. (1996). Influence of Rising Atmospheric CO2 and Phosphorus Nutrition on the Grain Yield and Quality of Rice (Oryza sativa cv. Jarrah). Australian Journal of Plant Physiology, 21(3), 281–292. https://doi.org/10.1071/PP9940281
**Shi et al 2022**	Shi, X., Shen, J., Niu, B., Lam, S. K., Zong, Y., Zhang, D., Hao, X., & Li, P. (2022). An optimistic future of C4 crop broomcorn millet (Panicum miliaceum L.) for food security under increasing atmospheric CO2 concentrations. PeerJ, 10. https://doi.org/10.7717/peerj.14024
**Singh et al 2013**	Singh, S., Bhatia, A., Tomer, R., Kumar, V., Singh, B., & Singh, S. D. (2013). Synergistic action of tropospheric ozone and carbon dioxide on yield and nutritional quality of Indian mustard (Brassica juncea (L.) Czern.). Environmental Monitoring and Assessment, 185(8), 6517–6529. https://doi.org/10.1007/s10661-012-3043-9
**Singh et al 2014**	Singh, S. K., Reddy, V. R., Fleisher, D. H., & Timlin, D. J. (2014). Growth, nutrient dynamics, and efficiency responses to carbon dioxide and phosphorus nutrition in soybean. Journal of Plant Interactions, 9(1), 838–849. https://doi.org/10.1080/17429145.2014.959570

**Singh et al 2016**	Singh, S. K., Barnaby, J. Y., Reddy, V. R., & Sicher, R. C. (2016). Varying Response of the Concentration and Yield of Soybean Seed Mineral Elements, Carbohydrates, Organic Acids, Amino Acids, Protein, and Oil to Phosphorus Starvation and CO2 Enrichment. Frontiers in Plant Science, 7, 1967. https://doi.org/10.3389/fpls.2016.01967
**Singh et al 2019**	Singh, S. K., Barnaby, J. Y., Reddy, V. R., & Sicher, R. C. (2019). Potassium Deficiency Influences Soybean Seed Mineral Compositions and Metabolic Profiles across CO2. American Journal of Plant Sciences, 10(12), 2113–2133. https://doi.org/10.4236/ajps.2019.1012149
**Soares et al 2019**	Soares, J., Deuchande, T., Valente, L. M. P., Pintado, M., & Vasconcelos, M. W. (2019). Growth and Nutritional Responses of Bean and Soybean Genotypes to Elevated CO2 in a Controlled Environment. Plants, 8(11), 465. https://doi.org/10.3390/plants8110465
**Soares et al 2021**	Soares, J. C., Zimmermann, L., Zendonadi dos Santos, N., Muller, O., Pintado, M., & Vasconcelos, M. W. (2021). Genotypic variation in the response of soybean to elevated CO2. Plant-Environment Interactions, 2(6), 263–276. https://doi.org/10.1002/pei3.10065
**Soba et al 2020**	Soba, D., Shu, T., Runion, G. B., Prior, S. A., Fritschi, F. B., Aranjuelo, I., & Sanz-Saez, A. (2020). Effects of elevated [CO2] on photosynthesis and seed yield parameters in two soybean genotypes with contrasting water use efficiency. Environmental and Experimental Botany, 178, 104154. https://doi.org/10.1016/j.envexpbot.2020.104154
**Ujiie et al 2019**	Ujiie, K., Ishimaru, K., Hirotsu, N., Nagasaka, S., Miyakoshi, Y., Ota, M., Tokida, T., Sakai, H., Usui, Y., Ono, K., Kobayashi, K., Nakano, H., Yoshinaga, S., Kashiwagi, T., & Magoshi, J. (2019). How elevated CO2 affects our nutrition in rice, and how we can deal with it. PLOS ONE, 14(3), e0212840. https://doi.org/10.1371/journal.pone.0212840
**Verrillo et al 2017**	Verrillo, F., Badeck, F.-W., Terzi, V., Rizza, F., Bernardo, L., Di Maro, A., Fares, C., Zaldei, A., Miglietta, F., Moschella, A., Bracale, M., & Vannini, C. (2017). Elevated field atmospheric CO2 concentrations affect the characteristics of winter wheat (cv. Bologna) grains. Crop and Pasture Science, 68(8), 713. https://doi.org/10.1071/CP17156
**Wang et al 2014**	Wang, Y., Song, Q., Frei, M., Shao, Z., & Yang, L. (2014). Effects of elevated ozone, carbon dioxide, and the combination of both on the grain quality of Chinese hybrid rice. Environmental Pollution, 189, 9–17. https://doi.org/10.1016/j.envpol.2014.02.016
**Wang et al 2020**	Wang, J., Li, L., Lam, S. K., Liu, X., & Pan, G. (2020). Responses of wheat and rice grain mineral quality to elevated carbon dioxide and canopy warming. Field Crops Research, 249, 107753. https://doi.org/10.1016/j.fcr.2020.107753
**Wang et al 2022**	Wang, X., Li, X., Zhong, Y., Blennow, A., Liang, K., & Liu, F. (2022). Effects of elevated CO2 on grain yield and quality in five wheat cultivars. Journal of Agronomy and Crop Science, 208(5), 733–745. https://doi.org/10.1111/jac.12612
**Wang et al 2023**	Wang, D., Ziska, L. H., Xu, X., Tao, Y., Zhang, J., Liu, G., Cai, C., Song, L., & Zhu, C. (2023). Adapting rice to rising atmospheric carbon dioxide: A preliminary GMO approach to maintain nutritional integrity. European Journal of Agronomy, 144, 126766. https://doi.org/10.1016/j.eja.2023.126766
**Wei et al 2018**	Wei, Z., Du, T., Li, X., Fang, L., & Liu, F. (2018). Interactive Effects of Elevated CO2 and N Fertilization on Yield and Quality of Tomato Grown Under Reduced Irrigation Regimes. Frontiers in Plant Science, 9. https://www.frontiersin.org/articles/10.3389/fpls.2018.00328
**Wei et al 2021**	Wei, L., Wang, W., Zhu, J., Wang, Z., Wang, J., Li, C., Zeng, Q., & Ziska, L. H. (2021). Responses of rice qualitative characteristics to elevated carbon dioxide and higher temperature: Implications for global nutrition. Journal of the Science of Food and Agriculture, 101(9), 3854–3861. https://doi.org/10.1002/jsfa.11021
**Wheeler et al 1997**	Wheeler, R. M., Mackowiak, C. L., Stutte, G. W., Yorio, N. C., & Berry, W. L. (1997). Effect of elevated carbon dioxide on nutritional quality of tomato. Advances in Space Research, 20(10), 1975–1978. https://doi.org/10.1016/S0273-1177(97)00263-9

**Wroblewitz et al 2013**	Wroblewitz, S., Hüther, L., Manderscheid, R., Weigel, H.-J., Wätzig, H., & Dänicke, S. (2013). The effect of free air carbon dioxide enrichment and nitrogen fertilisation on the chemical composition and nutritional value of wheat and barley grain. Archives of Animal Nutrition, 67(4), 263–278. https://doi.org/10.1080/1745039X.2013.821781
**Wu et al 2004**	Wu, D.-X., Wang, G.-X., Bai, Y.-F., & Liao, J.-X. (2004). Effects of elevated CO2 concentration on growth, water use, yield and grain quality of wheat under two soil water levels. Agriculture, Ecosystems & Environment, 104(3), 493–507. https://doi.org/10.1016/j.agee.2004.01.018
**Yadav et al 2019**	Yadav, A., Bhatia, A., Yadav, S., Kumar, V., & Singh, B. (2019). The effects of elevated CO2 and elevated O3 exposure on plant growth, yield and quality of grains of two wheat cultivars grown in north India. Heliyon, 5(8). https://doi.org/10.1016/j.heliyon.2019.e02317
**Yamakawa et al 2004**	Yamakawa, Y., Saigusa, M., Okada, M., & Kobayashi, K. (2004). Nutrient uptake by rice and soil solution composition under atmospheric CO2 enrichment. Plant and Soil, 259(1), 367–372. https://doi.org/10.1023/B:PLSO.0000020988.18365.b5
**Yang et al 2007**	Yang, L., Wang, Y., Dong, G., Gu, H., Huang, J., Zhu, J., Yang, H., Liu, G., & Han, Y. (2007). The impact of free-air CO2 enrichment (FACE) and nitrogen supply on grain quality of rice. Field Crops Research, 102(2), 128–140. https://doi.org/10.1016/j.fcr.2007.03.006
**Zheng et al 2020**	Zheng, G., Chen, J., & Li, W. (2020). Impacts of CO2 elevation on the physiology and seed quality of soybean. Plant Diversity, 42(1), 44–51. https://doi.org/10.1016/j.pld.2019.09.004
**Zhu et al 2018**	Zhu, C., Kobayashi, K., Loladze, I., Zhu, J., Jiang, Q., Xu, X., Liu, G., Seneweera, S., Ebi, K. L., Drewnowski, A., Fukagawa, N. K., & Ziska, L. H. (2018). Carbon dioxide (CO2) levels this century will alter the protein, micronutrients, and vitamin content of rice grains with potential health consequences for the poorest rice-dependent countries. Science Advances, 4(5), eaaq1012. https://doi.org/10.1126/sciadv.aaq1012
**Ziska et al 1997**	Ziska, L. H., Namuco, O., Moya, T., & Quilang, J. (1997). Growth and Yield Response of Field-Grown Tropical Rice to Increasing Carbon Dioxide and Air Temperature. Agronomy Journal, 89(1), 45–53. https://doi.org/10.2134/agronj1997.00021962008900010007x
